# Targeted bacterial conjugation mediated by synthetic cell-to-cell adhesions

**DOI:** 10.1093/nar/gkac1164

**Published:** 2022-12-13

**Authors:** Marta Robledo, Beatriz Álvarez, Ana Cuevas, Sheila González, David Ruano-Gallego, Luis Ángel Fernández, Fernando de la Cruz

**Affiliations:** Intergenomics Group, Instituto de Biomedicina y Biotecnología de Cantabria (IBBTEC), CSIC-Universidad de Cantabria, 39011, Santander, Spain; Biomar Microbial Technologies, Parque Tecnológico de León, Armunia, León 24009, Spain; Department of Microbial Biotechnology, Centro Nacional de Biotecnología, Consejo Superior de Investigaciones Científicas (CNB-CSIC), Campus UAM Cantoblanco, 28049 Madrid, Spain; Intergenomics Group, Instituto de Biomedicina y Biotecnología de Cantabria (IBBTEC), CSIC-Universidad de Cantabria, 39011, Santander, Spain; Intergenomics Group, Instituto de Biomedicina y Biotecnología de Cantabria (IBBTEC), CSIC-Universidad de Cantabria, 39011, Santander, Spain; Department of Microbial Biotechnology, Centro Nacional de Biotecnología, Consejo Superior de Investigaciones Científicas (CNB-CSIC), Campus UAM Cantoblanco, 28049 Madrid, Spain; Department of Microbial Biotechnology, Centro Nacional de Biotecnología, Consejo Superior de Investigaciones Científicas (CNB-CSIC), Campus UAM Cantoblanco, 28049 Madrid, Spain; Intergenomics Group, Instituto de Biomedicina y Biotecnología de Cantabria (IBBTEC), CSIC-Universidad de Cantabria, 39011, Santander, Spain

## Abstract

Genetic interventions on microbiomes, for clinical or biotechnological purposes, remain challenging. Conjugation-based delivery of genetic cargo is still unspecific and limited by low conjugation rates. Here we report an approach to overcome these problems, based on a synthetic bacterial adhesion system. Mating assemblers consist on a synthetic adhesion formed by the expression on the surface of donor and target cells of specific nanobodies (Nb) and their cognate antigen (Ag). The Nb–Ag bridge increased 1–3 logs transfer of a variety of plasmids, especially in liquid media, confirming that cell-cell docking is a main determinant limiting mating efficiency. Synthetic cell-to-cell adhesion allows efficient conjugation to targeted recipients, enhancing delivery of desired genes to a predefined subset of prey species, or even specific pathogenic strains such as enterohemorrhagic *Escherichia coli* (EHEC), within a bacterial community. The synthetic conjugation enhancer presented here optimizes plasmid delivery by selecting the target hosts with high selectivity.

## Graphical Abstract

**Graphical Abstract:**
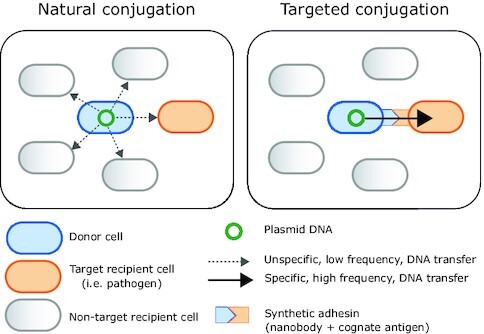
In this work, we convert bacterial conjugation into an effective target-driven DNA-delivery tool by engineering synthetic adhesins.

## INTRODUCTION

Controlled manipulation of the myriad of wild bacteria that dwell in natural ecosystems is at the cutting edge of biotechnology. Achieving targeted interventions would result in multiple clinical and environmental applications. To meet this challenge, DNA vectors encoding desired skills (therapeutics, bioremediators…) should effectively deliver their cargo to the targeted microorganisms. Conjugation, which enables natural transfer of genetic material through direct cell-to-cell contact, is a most suitable entry point for *in situ* bacterial genome engineering through mobile genetic elements (1). Plasmids have driven the development of a plethora of genetic engineering applications. Besides, they play important roles in systems and synthetic biology as genetic platforms to store, modify and transfer genetic information. Plasmids are autoreplicative genetic devices. Many of them naturally conjugate their DNA to recipient bacteria by encoding their own conjugative machinery. As a consequence, they have been engineered as tools for a variety of purposes such as gene expression vectors, mutagenic agents and, most recently, genetic modification of undomesticated microbial communities in soil (2) or in the animal gut microbiome (3–5).

However, tractable *in situ* interventions on natural bacterial populations must show efficiency and specificity. Conjugation is constrained by the need for direct cell-cell contact, and recognition of specific receptors in the recipient cells is needed to ensure specific DNA uptake (6,7). A better understanding of the intricate conjugation mechanism is essential not only to curb antibiotic resistance dissemination, but also to optimize the biotechnological applications described above. During natural conjugation, contact of donor to recipient cells is initially mediated by external appendages known as conjugative pili (8) that contain terminal adhesins (9). Conjugative pili are assembled by type IV secretion systems (T4SS) encoded within the conjugative plasmids (10). These pili can be long and flexible, or short and rigid (11). Plasmids that determine flexible pili (e.g. Inc F, H and I), transfer equally well in liquid as in solid surfaces, while those encoding rigid pili, such as Inc N, P and W, conjugate at rates 2–4 orders of magnitude higher on solid surfaces (12,13). Despite progress in the identification of conjugation components needed for DNA transfer (11,14), key plasmid transfer and reception mechanisms remain obscure.

Here, we aimed to fill these knowledge gaps while, at the same time, developing a technology to pave the way to *in situ* microbiome control. We aim to do it by constructing synthetic bridges that mediate efficient conjugation from a donor cell to a targeted recipient cell. To this end, we use synthetic adhesins (15), composed of the N-terminal outer membrane (OM)-anchoring domain of intimin, called Neae (16,17), fused to a nanobody (Nb). Nbs are recombinant single-domain antibody fragments derived from the VHH domains of heavy chain-only antibodies naturally found in camelids (18). Despite their small size (ca. 15 kDa) and simple structure, Nbs bind their cognate antigen (Ag) with great specificity and affinity. The surface display of Nbs with intimin Neae domain (19,20) mediated rapid and highly specific attachment of *Escherichia coli* bacteria to different cells expressing the recognized Ag on their surface, including mammalian tumor cells (15) and other *E. coli* bacteria (21,22). Furthermore, *Enterobacter cloacae* expressing Nbs deplete target *E. coli* cells producing the cognate Ag via type VI secretion system in liquid conditions (23).

In this work, we engineered synthetic cell-to-cell adhesions between donor and recipient bacteria by displaying a Nb or its complementary Ag pair on their surface. *E. coli* bacteria displaying Nbs or Ags were used as donor or recipient cells in a series of conjugation experiments, shedding light on mechanisms controlling DNA spread among microorganisms. The intercellular bridges assembled by Nb–Ag pairing increased conjugation frequencies 1–3 logs in liquid media of conjugative plasmids with rigid pili as N-, P- or W-pili, reaching yields similar to those obtained on solid surfaces. We also show that synthetic cell-to-cell adhesions mediate high-frequency plasmid mobilization even in mating conditions in which the target cells are a minority of the bacterial population, thus demonstrating the specificity of this system. Lastly, we provide proof-of-principle for targeted conjugation to a pathogenic strain of enterohemorrhagic *E. coli* (EHEC) (24). These experiments also revealed the importance of selecting a synthetic adhesin recognizing an accessible Ag on the surface of the pathogen for effective targeting. Taken together, our data unveil the potential of synthetic adhesins as precise nanotools for programmable DNA delivery through conjugation in complex microbial populations. In addition, our work highlights the importance of specific adhesion mechanisms between bacteria as driving forces for the spread of conjugative antibiotic-resistance plasmids in nature.

## MATERIALS AND METHODS

### Bacterial strains, media and growth conditions

The *E. coli* strains and plasmids used in this work are listed in Supplementary Tables S1 and S2, respectively. For plasmid propagation and cloning procedures, strains DH10B-T1R and BW25141 (for the pir-dependent plasmids) were used. MG1655-derivative EcM1 and its derivatives were used for constitutive expression of the synthetic adhesins, the TirM and EHEC full-length Intimin from the chromosome. A BW27783 spontaneous mutant resistant to nalidixic acid (BW-Nx^R^) was obtained by plating out BW27783 on LB-agar containing the antibiotic (spontaneous mutants did not show significant defects in growth).

Bacteria were statically grown overnight in Luria−Bertani (LB) liquid medium and agar plates (1.5% w/v) at 37 °C, unless otherwise indicated. When appropriate, antibiotics were added at the following concentrations: ampicillin sodium salt (Ap; 100 μg/ml), chloramphenicol (Cm; 25 μg/ml), kanamycin (Km; 25 μg/ml); nalidixic acid (Nx; 20 μg/ml), rifampicin (Rif; 50 μg/ml), streptomycin (Sm; 300 μg/ml). Plac- (Nbs) and Ptac-driven (*grlA*) expression were induced by adding 0.1 mM and 0.02 mM IPTG to culture media, respectively.

### Plasmids, constructs and primers

A list of the plasmids and primers used in this study can be found in Supplementary Tables S2 and S3, respectively. Standard cloning protocols of DNA digestion with restriction enzymes and ligation (Sambrook and Russel, 2001) were followed for building the constructs. All the PCR amplifications for cloning purposes were carried out with the Herculase II Fusion DNA polymerase (Agilent Technologies). The DNA sequence of all the constructs was determined by the chain-termination Sanger method (Macrogen). For IPTG-inducible expression of the TirM peptide from EHEC on the cell surface, the plasmid pTirMA carrying a C-EhaA TirM fusion was used. To build this plasmid, the TirM was amplified by PCR with the primers F_SfiI_TirM and R_NotI_TirM (Supplementary Table S3) using the plasmid pET28a-TirM_EHEC_ (25) as template. The TirM amplicon was cloned into pHEA using the restriction enzymes SfiI and NotI, resulting in the plasmid pTirMA. For the constitutively expression of the C-EhaA TirM fusion from the chromosome, the strain EcM1*flu::TirMA* was generated as described below using the suicide plasmid pGEfluTirMA, a derivative of pGE. This plasmid was built by excising the fusion from the plasmid pTirMA and cloned into pGEflu-SAgfp (15) using the restriction enzymes XbaI and HindIII. The plasmid pGERecombTS-intEHEC, used to generate the strain EcM1-NL-intEHEC that produces the intimin of EHEC, was derived from the plasmid pGETS. Briefly, the DNA sequence coding the C-terminal part of the intimin was amplified with the primers 5′ XhoI HR int and 3′ SpeI HindIII int (Supplementary Table S3) using genomic DNA isolated from EHEC EDL933 stx as template, and cloned in the backbone of pGETSfluPtac-eLEE5 cut with the enzymes XhoI and SpeI. Then, an apramycin resistant cassette was amplified with the primers 5'_HindIII_FRT_Apra and 3'_SpeI_FRT_Apra (Supplementary Table S3) and cloned downstream the C-ter intimin using the enzymes HindIII and SpeI, obtaining the plasmid pGERecombTS-intEHEC. The nanobody IB10 was obtained from the plasmid pEHlyA5-IB10 (25) by PCR amplification with the primers VHH-SfiI2 and VHH-NotI2, digested with SfiI and NotI and cloned into pNVgfp previously cut with the same enzymes, and thus replacing the Nb anti-gfp. The resulting plasmid was named pNeae-IB10. The plasmids pNVtir1 and pNeae-IB10 were used for expression of the anti-TirM and the anti-Intimin Nb IB10 on the bacterial surface, respectively. Bacteria carrying pHEA and pNVgfp, were used as control. Plasmid pSA10_GrlA-6his was introduced into EHEC EDL933 stx- or its derivative strains for IPTG-inducible expression of *grlA*.

### Generation of the Strains EcM1TirMA and EcM1-NL-intEHEC

For the generation of the strain EcM1TirMA, the cassette with the fusion C-EhaA TirM under the control of the PN25 promoter was integrated into the *flu* site of the strain EcM1 using a marker-less genome edition strategy as previously described (15). Following this strategy, the strain EcM1 was transformed with the plasmid pACBSR that contains the genes coding for I-SceI and λ Red proteins under the control of the PBAD promoter that is inducible with L-arabinose. The strain EcM1 with pACBSR was then electroporated with the pir-dependent plasmid pGEfluTirMA. Since EcM1 lacks the pir protein, the plasmid pGEfluTirMA with homology regions flanking the *flu* site and I-SceI restriction sites could not replicate and got integrated into the genome of this strain. The co-integrants were selected by plating on LB agar with Km and Cm. Resistant colonies were grown overnight at 37 °C with shaking (250 rpm) in LB supplemented with Km and Cm. Next day, cultures were diluted 1:100 in LB with Cm and incubated under the same conditions until reached OD_600_ of 0.4–0.6, when 0.4% l-arabinose was added to induce the production of I-SceI and λ Red proteins for promoting the second homology recombinant event. Cultures were further incubated for 5 h, and then, spread onto LB agar with Cm. The obtaining colonies were streaked on LB agar plates with and without Km to confirm that they are susceptible to Km due to the loss of vector sequences. The integration of the fusion C-EhaA TirM was assessed by colony PCR using the primers F_flu_int and R_NotI_TirM (Supplementary Table S3). The last step was curing the pACBSR from the final strain by passaging the cultures in LB without antibiotic.

The strain EcM1-NL-intEHEC was generated modifying a EcM1 strain harboring the anchoring domain of intimin under the control of the Ptac promoter inserted in the *flu* locus by inserting the C-terminal part of intimin, and thus, creating a full-length intimin. To do that, the parental strain with the plasmid pACBSR was transformed with the plasmid pGERecombTS-intEHEC that contains the thermosensitive origin of replication pSC101-ts, and HRs of the intimin and flu, and I-SceI restriction sites. The strain with both plasmids was grown overnight in LB with Cm and Km at 250 rpm and 30 °C, a permissive temperature for the pSC101-ts plasmids replication. Next day, the culture was diluted 1:100 in LB with Cm and Apra and grown until OD_600_ of 0.4–0.6, when 0.4% l-arabinose was added and the cultures were further incubated at 37 °C to avoid the replication of pGERecombTS-intEHEC. After 5 h, the cultures were plated on LB agar with Cm and Apra. The resulting colonies were tested for sensitivity to Km as above and the reconstruction of the full intimin in the flu site was confirmed by PCR using the primers Int_EHEC_seq and 3′ flu genome. As above, the plasmid pACBSR was cured from the final strains by passaging the cultures in LB without antibiotic.

### Conjugation assays

Donor and recipient K-12 strains were grown overnight from single colonies with the appropriate antibiotics. Donor plus recipient strains were centrifuged for 5 min at 4000 × g and resuspended in appropriate volumes according to their OD_600_. The cell cultures, corresponding to an OD equivalent of 1 (0.5 for target conjugations), were mixed at 1:1 proportion, centrifuged and resuspended in 400 μl LB medium with 0.1 mM IPTG. Suspensions were either placed in a 24-well plate well (liquid conjugations) or centrifuged again, resuspended in 10 μl LB and extended on top of wells containing 1 mL of LB-agar with 0.1 mM IPTG (solid conjugations). Conjugation was allowed to proceed for 1 or 2 h at 37 °C without agitation. At this point, macroscopic aggregation of conjugative liquid matings was checked. Bacteria were resuspend in 1 ml PBS after mating, vortexed for 10 s and serial dilutions plated in triplicate on selective media. Conjugation and mobilization frequencies were estimated as the number of transconjugant cells receiving the plasmid per donor (T/D) or per recipient (T/R) as indicated.

Recipient EHEC strains were grown from single colonies for 6 h at 120 rpm in a flask with 10 mL of liquid LB with the corresponding antibiotics, inoculated in capped Falcon tubes (BD Biosciences) with 5 ml Dulbecco's Modified Eagle's Medium (DMEM), and incubated overnight at 37 °C in a CO_2_ incubator (static) to resemble infection conditions. EHEC and donor cultures were resuspended in appropriate volumes to an OD equivalent of 2, mixed in a 1:1 proportion, centrifuged and resuspended in 400 μl DMEM without IPTG (liquid conjugations) or centrifuged again, resuspended in 10 μl DMEM and extended on wells containing 1 mL of DMEM-agar without IPTG (solid conjugations). Plates were incubated 2 h at 37 °C in a CO_2_ incubator without agitation. Conjugation frequencies were estimated as described above.

### Cell aggregation assays

Liquid cultures of *E. coli* K-12 strains were separately grown overnight and mixed 1:1 as described before for liquid conjugations, resuspended in 1.9 ml LB medium with 0.1 mM IPTG and incubated at 37 °C without shaking. Triplicate 100-μl samples were withdrawn from the top of the cultures immediately following mixture, after 30-min and at 1h intervals from the top of each tube. Samples were transferred to 96-well assay plates and OD_600_ was measured on a TECAN microplate reader. Test tubes containing the original mixed samples were store at 37 °C and photographed after 24 h.

For the aggregation assays to EHEC strains, cultures were grown overnight as described in the previous section. K-12 and EHEC bacteria cultures were resuspended in PBS to an OD equivalent of 5, mixed in a 1:1 proportion, centrifuged and resuspended in 1 ml PBS. Mixtures were incubated at 37 °C without agitation and OD_600_ of 100-μl samples withdrawn from the top of the cultures was measured at different time intervals as described above.

### Fluorescence microscopy

Microscope images were taken from 2 h liquid conjugation matings. Donor cells carried the conjugative plasmid pAR106, producing GFP, while recipient cells were BWmKate, expressing a RFP upon IPTG-induction. This set up allows transconjugant visualization, which are cells expressing both fluorescence markers. After mating, 2 μl-samples were carefully taken from the bottom of the wells and placed onto 1.5% agarose pads, generated by stacking an adhesive frame (Frame-Seal Incubation Chamber, Bio-Rad) onto a microscope slide. The culture droplet was allowed to dry for ∼5 min before a coverslip was placed over the frame. Bacteria were examined by epifluorescence using a Leica AF6500 inverted microscope equipped with × 630 magnification using HCX PL S-APO 63× 1.3 oil objective and a 12-bit Andor iXon885 high-speed camera. Images were acquired by phase contrast in the green and red fluorescence channels. Filters used for fluorescence images were 562/40-nm excitation band pass and 641/75-nm emission for mKate and 482/18-nm excitation and 520/28-nm emission for GFP. Images were acquired using LAS AF software (Leica) and edited with FIJI (Image J software).

### Statistical analysis

Conjugation and mobilization frequencies means and SD were calculated using decimal logarithms of log-normally distributed data. Data was represented using the Prism biostatics software (GraphPad, San Diego, CA). Mean comparison between samples and control conditions was carried out by unpaired Student's *t*-test.

## RESULTS

### Genetically encoded synthetic adhesins for programming bacterial conjugation

**Figure 1.**
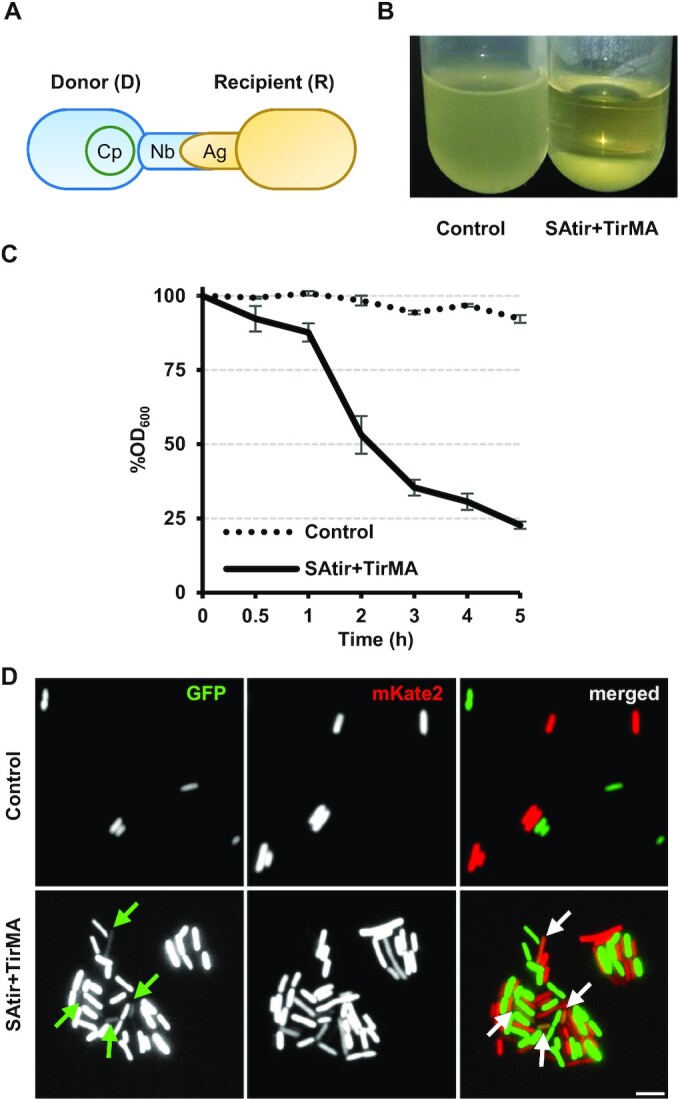
Genetically encoded synthetic adhesins for programming bacterial conjugation. (**A**) General scheme showing donor (blue; carrying a conjugative plasmid, Cp) and recipient (yellow) *E. coli* cells. Their outer membranes are anchored because the donor expresses a surface-bound Nb as synthetic adhesin (SAtir), while the recipient expresses its complementary antigen (TirMA). (**B**) Macroscopic aggregation observed when RP4 donor bacteria constitutively expressing TirMA are mixed in 1:1 ratio with recipient cells carrying either pNVgpf (left; control) or pNVtir1 (right; SAtir + TirMA). (**C**) Relative OD_600_ of control and SAtir + TirMA cultures at different time points (measures taken from upper 100 μl of cultures). (**D**) Fluorescence microscopy of 1:1 matings of control or SAtir + TirMA cells. Donors carry a RP4 derived conjugative low-copy plasmid expressing a green fluorescent protein gene (pAR106), while recipient cells are the strain BWmKate2 encoding the red fluorescent protein mKate2, allowing visualization of transconjugants co-expressing GFP and RFP (arrows).

We hypothesized that a conjugative donor cell expressing a synthetic adhesin (i.e. displaying a Nb of known specificity) would specifically adhere to recipient cells displaying the cognate Ag, thus possibly increasing conjugation rates in liquid media. To test this, we generated *E. coli* donor and recipient cells displaying a Nb–Ag pair. As a model, we used a synthetic adhesin called SAtir (Supplementary Table S1), displaying a Nb binding the TirM domain of the translocated intimin receptor (Tir) from EHEC (15,19) (Figure 1A). Display of TirM Ag on *E. coli* surface was achieved by its fusion to the autotransporter domain of EhaA (19,26), and the resulting fusion was called TirMA (Supplementary Table S1). To address system versatility, SAtir and TirM were expressed from either multicopy plasmids with an IPTG-inducible P*lac* promoter (pNVtir1 and pTirMA, respectively; Supplementary Table S2) or from single-copy constructs expressed by a constitutive promoter integrated in the chromosome of *E. coli* K-12 (strains EcM1SAtir and EcM1TirMA, respectively; Supplementary Table S1). It has been previously shown that Nb display with intimin or EhaA autotransporter domains does not affect the growth and the viability of *E. coli* from inducible plasmids nor when constitutively expressed from the chromosome (15,19). Further, constitutive expression of synthetic adhesins from integrated constructs is maintained throughout multiple generations both *in vitro* and *in vivo* (in mice) without selection pressure (15,19).

We first tested cell adhesion by mixing stationary phase liquid cultures at 1:1 D:R ratio. Mixtures of bacteria displaying the Nb–Ag pair SAtir-TirM formed macroscopic aggregates and settled down at the bottom of the test tube, whereas bacteria displaying this Ag remained non-aggregated when mixed with bacteria displaying a control synthetic adhesin binding the green fluorescent protein (GFP), named SAgfp (15,19) (Figure 1B). Quantification of the optical density at 600 nm (OD_600_) in the upper 0.1 ml of these cultures along time showed that bacterial mixtures with SAtir-TirM pairs started to aggregate 30 min after mixing and ∼80% of the bacteria had settled down in 5 h (Figure 1C).

The spatial distribution of the mating assemblies was inspected using donor cells with a RP4-derived conjugative plasmid encoding GFP and recipient cells expressing mKate2 red fluorescent protein (RFP) (27). Fluorescence microscopy of control mating mixtures showed a uniform suspension of donor and recipient cells. On the other hand, matings with SAtir-TirM pair resulted in aggregates having a mesh-like pattern of green and red cells (Figure 1D). Plasmid transfer, manifested by transconjugant cells co-expressing GFP and RFP, could be spotted in some bacteria of these aggregates (labeled with arrows in Figure 1D).

### Synthetic cell-to-cell adhesions increase conjugation frequencies in liquid media of IncP, IncN and IncW plasmids encoding rigid pili

To check whether formation of synthetic cell-to-cell adhesions enhance conjugation, we performed matings of *E. coli* donor cells displaying the Ag TirM (strain EcM1TirMA) with recipient cells displaying the Nb SAtir (strain BW27783 + pNVtir1) or the control SAgfp (strain BW27783 + pNVgfp). Conjugation frequencies of different plasmids (see below) were calculated as the number of transconjugants per donor (T/D) 2 h after donor and recipient cells were mixed at 1:1 ratio.

**Figure 2.**
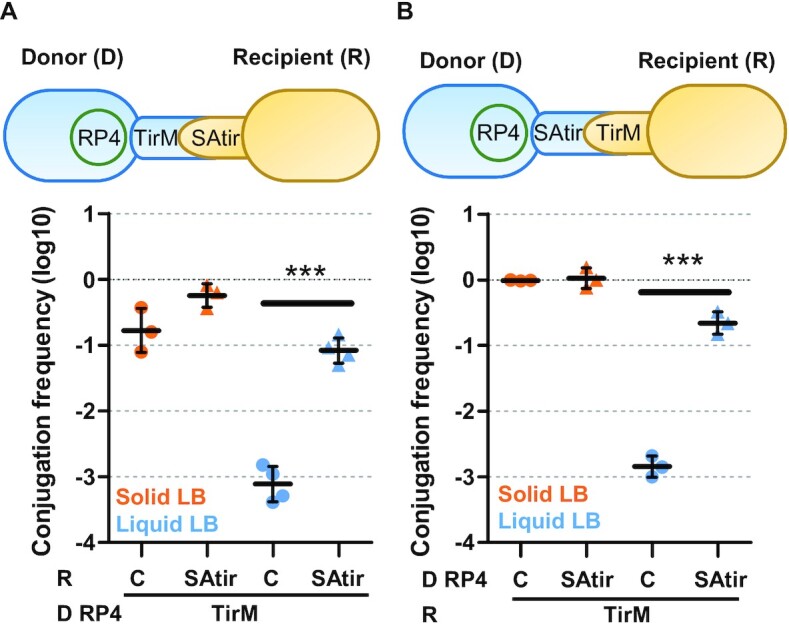
The synthetic adhesion system is composable and increases conjugation frequencies in liquid media. Schematic representation of the experiments and conjugation frequencies (Transconjugants/Donor) of conjugative plasmid RP4 measured by plate conjugation after grown 2 h in either solid (orange points) or in liquid (blue) LB media. (**A**) Donor (D) bacteria were EcM1flu::TirMA, constitutively expressing TirM Ag on the bacterial surface. Recipient (R) cells were *E.coli* BW27783 carrying either pNVgfp (Control; C) or pNVtir1 (SAtir) expressing an anti-TirM nanobody upon induction with 0.1 mM IPTG. (**B**) Donor bacteria were *E. coli* BW27783 with pNVgpf or pNVtir1 and recipient cells were EcM1flu::TirMA. Each point represents the result of one independent experiment shown in logarithmic scale; horizontal and vertical bars represent the mean ± SD of each group of data. ***P < 0.001 by unpaired Student's *t*-test.

IncP (pRL443 here onwards referred to as RP4) is a broad-host plasmid encoding rigid pili that conjugate more efficiently on solid surfaces than in liquid cultures. Conjugation frequencies on solid surfaces were high (ca. 10^−1^) and did not significantly vary in the presence of the displayed Nb–Ag pairs (Figure 2A), indicating no negative effects of their expression on the assembly of these conjugative pili. In contrast, synthetic donor-to-recipient adhesion increased conjugation of these rigid-pili plasmids in liquid media by ca. 2 logs compared to controls, reaching frequencies close to those obtained on solid surfaces (Figure 2A). A similar increase in conjugation in liquid was observed in a reciprocal mating with donor cells expressing the adhesin SAtir and recipient cells expressing the Ag TirM (Figure 2B), demonstrating that the synthetic adhesion system is composable and improves plasmid transfer rates in liquid regardless of the expression system used and whether the synthetic adhesion components are chromosomally or plasmid-encoded.

We systematically analyzed this increase in liquid conjugation efficiency using alternative rigid pili-encoding plasmids like pKM101 (IncN; Supplementary Figure S1) and R388 (IncW, Supplementary Figure S2); and observed significative differences, e.g. 100-fold increase for RP4 or 28-fold for R388 (Table 1). This increment was more pronounced after 2 h conjugation compared to 1 h (Supplementary Table S4). Together, these results indicate that donor-recipient cell-to-cell adhesions mediated by specific Nb–Ag interaction constitute a suitable and versatile tool to increase conjugation in liquid media.

**Table 1.** Transfer frequency of a set of prototype conjugative and mobilizable plasmids increases with the anchoring system. Transfer frequencies are expressed as average fold increases relative to controls without anchoring system and were determined as described in Figure 2

					Relative transfer frequency	
Plasmid name	Inc	Pilus type	MOB family	Mating scheme	Solid	Liquid
RP4	P1α	Rigid	F11	Figure 2	3.41	107.59***
pKM101	N	Rigid	F11	Supplementary Figure S1	0.68	47.69***
R388	W	Rigid	F11	Supplementary Figure S2	2.75	28.09
R1	F	Flexible	F12	Supplementary Figure S4A	2.26	0.83
R1drd	F	Flexible	F12	Supplementary Figure S4B	1.15	1.16
RP4	P1α	Rigid	F11	Supplementary Figure S5A	1.28	69.16***
RSF1010	Q1	none	Q11	Supplementary Figure S5A	1.16	1511.74***
RSF1010	Q1	none	Q11	Figure 3A	7.08**	24.62***
RP4	P1α	Rigid	F11	Figure 4	1.22	55.63***
RSF1010	Q1	none	Q11	Figure 4	1.69	822.18***

Inc, plasmid incompatibility group (58). MOB, MOB group (59). **P < 0.01, ***P < 0.001, by unpaired Student's *t*-test.

### Synthetic adhesion of donor and recipient cells does not complement lack of pili adhesin for conjugation

Prevailing models of bacterial conjugation support that conjugative T4SS pili provide essential contacts between donor and recipient cells. Conjugative pili are assembled by an hydrophobic protein pilin (VirB2 or TrwL in plasmid R388), with the essential incorporation of adhesin molecules (VirB5/TrwJ) at the distal end (28). We investigated whether an insertion mutant inactivating the terminal adhesin TrwJ of plasmid R388 (R388 *trwJ*) could be complemented by synthetic donor-to-recipient cells adhesion. As expected, conjugation of R388 *trwJ* plasmid was very low (close to detection limit) in liquid, but quantifiable on solid medium (Supplementary Figure S3). Notably, synthetic adhesion of donor and recipient cells mediated by the Nb–Ag pair did not revert the lack of functional TrwJ in liquid conjugations (Supplementary Figure S3). Therefore, a functional conjugative pilus with its terminal adhesin is essential for conjugation to occur, even in the presence of synthetic adhesins.

### Synthetic adhesins are more efficient conjugation enhancers than IncF liquid-mating plasmids

We next assessed the effect of donor-to-recipient cell adhesion on the conjugation of the *E. coli* IncF plasmids R1, and its derepressed version R1drd19, which produce flexible-pili F capable of liquid conjugation (29). As observed in Supplementary Figure S4, plasmid R1 exhibited conjugation frequencies markedly reduced with respect to R1drd19, due to its repressed conjugation system (30). In contrast to IncP, IncW or IncN plasmids, in which synthetic adhesins increased conjugation frequencies in liquid, mating of plasmid R1 and R1drd remained unperturbed by the presence of Nb–Ag interaction between donor and recipient cells (Supplementary Figure S4; Table 1), demonstrating the lack of effect of the synthetic system on flexible pili-encoding IncF plasmids. Thus, flexible conjugative pili appear to be sufficient to mediate adhesion to recipient cells in liquid environments and synthetic adhesins did not improve these natural systems. Results also indicated that repressed pilus status cannot be turned on by the synthetic system.

To check whether the co-residence of IncF plasmids would enhance conjugation frequencies of rigid-pili plasmids in liquid matings, we chose the IncW plasmid R388, which showed the lowest increase in liquid matings with the synthetic adhesion system (Table 1). As previously reported (31,32), co-residence of R1drd19 in donor cells negatively affected R388 transfer in surface matings due to fertility inhibition. However, when the experiments were carried out by liquid conjugation, the transfer of R388 significantly increased 1 log in the presence of co-resident plasmid R1drd19 (Supplementary Figure S2). We then checked whether R1drd19 pili-mediated anchoring could increase conjugation in liquid matings at both ends (i.e. if it also occurs when facilitating plasmid is present in recipient cells). Results showed that, in comparison with control matings, the transfer of R388 when R1drd is present in recipient cells was lower or remained unchanged in both solid and liquid matings. Therefore, R1drd conjugation system facilitates cell anchoring and liquid mating of the rigid-pili encoding plasmid R388 (∼1 log) only when both reside in donor cells, that is, the effect is unidirectional. In contrast, synthetic adhesions mediated by Nb–Ag pairs are composable and increased R388 plasmid transfer in liquid significantly more than the presence of IncF plasmid in Donor cells (Supplementary Figure S2).

### Synthetic adhesion system increases mobilization frequencies in liquid and solid media

Mobilizable plasmids need the machinery of a conjugative element (helper) co-resident in the cell. They transfer poorly in liquid media when helped by rigid-pili plasmids (33). To find out whether the synthetic adhesion system may increase mobilization frequencies, the transfer of the mobilizable plasmid RSF1010 by helper plasmid RP4 was analyzed in the presence of the synthetic adhesion system. Supplementary Figure S5A shows the frequencies of transference of RP4 and RSF1010 plasmids, on surface and liquid assays. To this end, *E. coli* cells carrying RSF1010 and the Ag-coding plasmid (pTirMA) were mated with cells constitutively expressing the Nb SAtir from the chromosome (EcM1SAtir) and containing the conjugative plasmid RP4 as depicted in the scheme. Similar to results in Figure 2, we observed that conjugation frequencies of RP4 significantly increased 69-fold in liquid media with the synthetic adhesion system (Supplementary Figure S5A, Table 1), almost reaching the yields obtained on surfaces.

In the absence of synthetic adhesion system, the transference of the mobilizable plasmid was ∼2–3 logs lower in liquid and surface matings than the conjugative frequencies found with plasmid RP4. However, liquid mixtures with specific adhesion (SAtir-TirM) caused an outstanding 1,512-fold increase in RSF1010 mobilization frequencies with respect to controls (Supplementary Figure S5A, Table 1). The mating system was exchanged by using bacteria carrying RSF1010 transformed with pNVtir1, encoding SAtir, or control plasmid pNVgfp and *E. coli* RP4+ cells constitutively displaying the Ag TirM (strain EcM1TirMA). The results obtained, shown in Supplementary Figure S5B, are consistent with those shown in Supplementary Figure S5A; further demonstrating that the synthetic adhesion system is composable.

**Figure 3.**
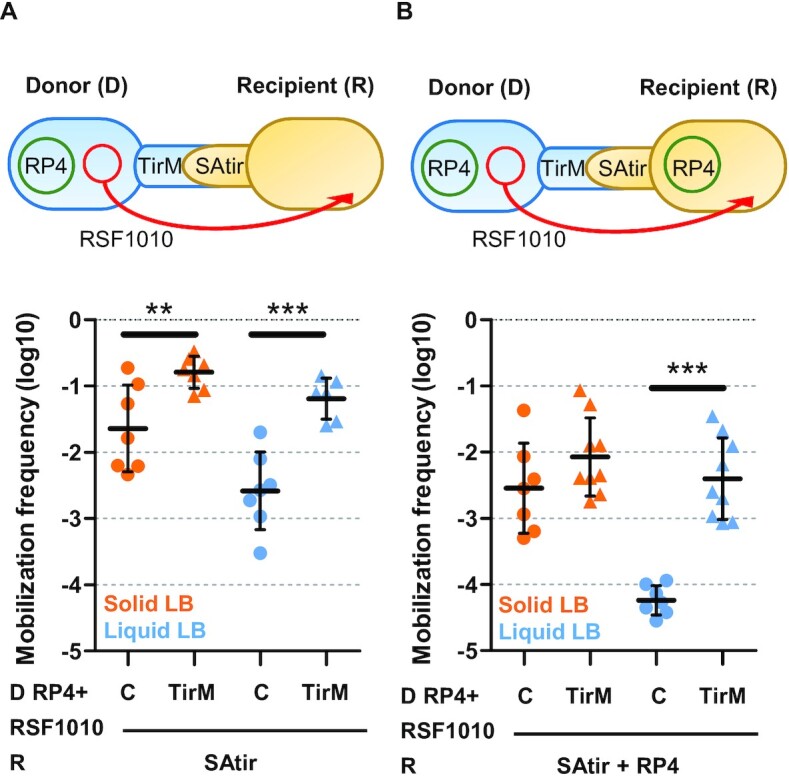
Synthetic adhesion increases mobilization frequencies and overcome entry exclusion. Schematic representations of the experiments and mobilization frequencies (Transconjugants/Recipient) shown in logarithmic scale of RSF1010 plasmids after grown 2 h. Donors (D) were either EcM1 (C) or EcM1flu::TirMA (TirM) strains carrying RP4 and RSF1010. (**A**) Recipients (R) were transformed with pNVtir1 (SAtir) alone. (**B**) Recipients (R) carry SAtir plus RP4, to resemble eex conditions. **P < 0.01, ***P < 0.001, by unpaired Student's *t*-test.

Plasmid mobilization by synthetic adhesion was also assessed using donor cells (displaying or not the TirM Ag) and carrying both the conjugative RP4 and the mobilizable RSF1010 plasmids mated with recipient cells expressing the SAtir (Figure 3A, scheme). Mobilization in liquid matings increased almost 2 log between cells having the cognate Nb–Ag contacts, overcoming the transfer frequency obtained in control solid matings (Figure 3A). Interestingly, in the presence of the corresponding Nb–Ag pair, mobilization frequencies of RSF1010 plasmid helped by RP4 significantly increased ∼1 log even on solid media (Figure 3A; Table 1). This was the first positive effect observed on solid matings, demonstrating that synthetic adhesion between D-R cells can also improve surface mating of mobilizable plasmids like RSF1010.

### Plasmid entry exclusion can be partially overcome by the synthetic adhesion system

Entry exclusion (eex) is a phenomenon described for most conjugative plasmids by which plasmids in recipient cells prevent the further entry of certain conjugative plasmids. Eex involves recognition of a conjugation machinery by a protein present in the inner membrane of the recipient cell (Trbk in RP4-like plasmids) (34). For instance, when RP4 is present in recipient cells, transfer of the mobilizable plasmid RSF1010 from donors carrying a co-resident RP4 decreases by the action of the eex protein TrbK (35).

We aimed to determine the effect on eex of the synthetic donor-recipient cell adhesion system. We assessed transfer of RSF1010 from donor (RP4+) cells to recipient cells harboring RP4 (eex+) or plasmid-free (eex−). In the absence of the synthetic adhesion system, eex on solid media lead to mobilization frequencies of RSF1010 that resembled those obtained in control liquid matings to RP4-free recipients (∼5 × 10^−3^ T/D; Figure 3A, B). Synthetic adhesion of donor and recipient cells significantly increased mobilization of RSF1010 compared to the controls even when recipient cells carried the RP4 plasmid responsible for eex in liquid matings (Figure 3B). These results further evidence the extraordinary increase of plasmid mobilization in liquid in the presence of the synthetic adhesion system, which could even partially overcome the inhibitory effect of eex. Interestingly, mobilization frequencies in liquid to recipients harboring a co-resident plasmid in Nb–Ag labelled cells is similar to those obtained when control plasmid-free cells are used as recipients without the adhesion system (Figure 3A, B).

### Synthetic adhesion enhances DNA transference in complex triparental mating schemes

**Figure 4.**
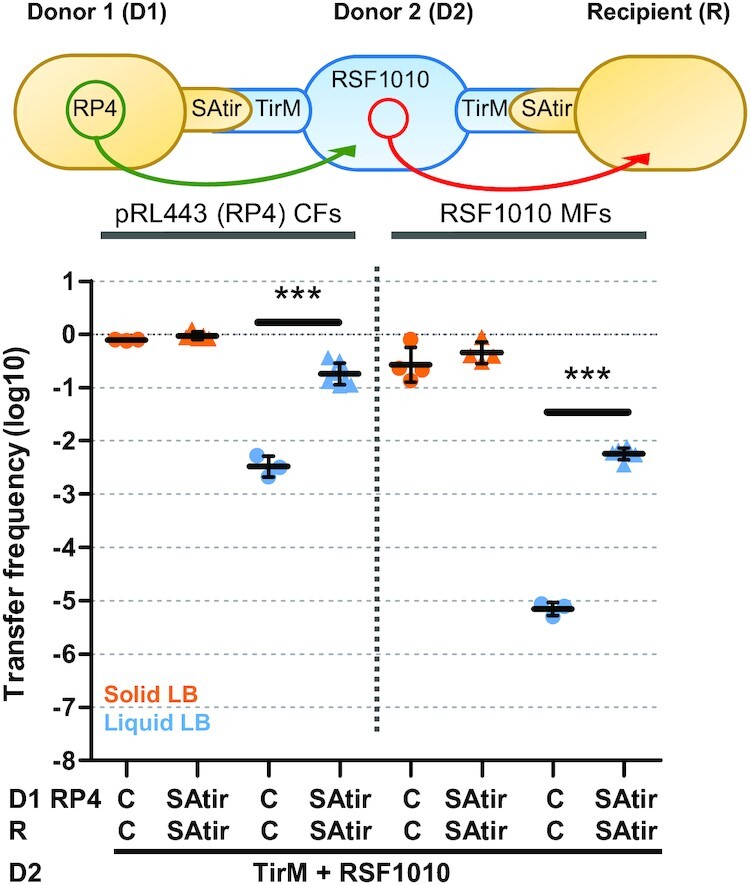
Synthetic adhesion enhances DNA tranference in complex triparental mating schemes. Schematic representations of the experiments, conjugation frequencies (CFs) and mobilization frequencies (MFs) (transconjugants/recipient) shown in logarithmic scale of RP4 and RSF1010 plasmids after grown 2 h. Donor 1 (D1) and recipients (R) both carried either pNVgfp (Control; C) or pNVtir1 (SAtir). Donor 2 (D2) cells were EcM1flu::TirMA plus RSF1010. D1 cells also carried RP4, which mobilizes RSF1010 upon conjugation to D2 cells. ***P < 0.001, by unpaired Student's *t*-test.

We sought to rationally design matings involving more than two cell types (Figure 4). In some experimental set-ups, plasmids are introduced in recipient cells by triparental mating with two donor cells: one strain carrying a conjugative plasmid (Donor 1; D1) and the other a mobilizable plasmid (Donor 2; D2) (36). In these cell mixtures, conjugative and mobilizable plasmids are present in different cells, so they do not need to stably coexist in the same strain. Thus, it seemed interesting to test the effect of the synthetic adhesion system on the transfer of mobilizable RSF1010 plasmid in liquid in the presence of a helper strain carrying a conjugative plasmid RP4 (D1). We achieved this by using D2 cells expressing Ag (TirM) and carrying the RSF1010 mobilizable plasmid to achieve adhesion to both D1 and recipient cells, both encoding the SAtir counterpart (Figure 4). Conjugation frequencies of RP4 from D1 to D2 cells and mobilization of RSF1010 from D2 to R cells remained invariable in the presence of the adhesins on solid media. Furthermore, mobilization of plasmid RSF1010 was low (∼10^−5^ T/R) in liquid assays. As expected, in the presence of the interacting Nb–Ag pair, a ∼55-fold increase was observed in the liquid transfer of RP4 whereas the increment of transfer for RSF1010 was ∼822-fold (Figure 4; Table 1). Permutations of the Nb–Ag constructs between mating cells also resulted in higher rates of transference for RP4 and RSF100 (Supplementary Figure S6), demonstrating the system composability.

### Synthetic adhesion system allows conjugation to specific recipient targets

**Figure 5.**
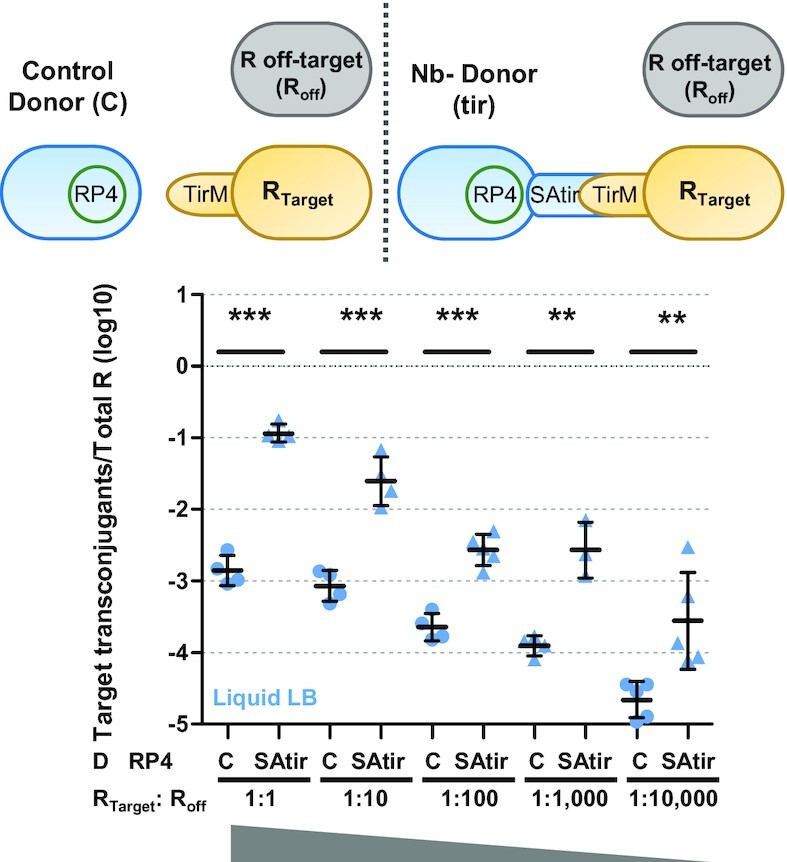
Synthetic adhesion allows conjugation to specific recipient targets. Schematic representation of the experiments and RP4 conjugation frequencies expressed as log10 of target transconjugants per total number of recipients (R) after grown 2 h in liquid LB medium. Donor bacteria were *E. coli* BW27783 cells carrying RP4 and either plasmid pNVgpf expresing SAgfp (Control; C) or pNVtir1 (SAtir). R were a mixture of target EcM1flu::TirMA cells (R_Target_), and non-target BWmKate2 cells (*R*_off-target_; *R*_off_) mixed at the indicated ratios (D and *R*_off-target_ cells were always at a 1:1 proportion). **P < 0.01, ***P < 0.001, by unpaired Student's *t*-test.

Plasmid spreading to a specific recipient target through conjugation in a mixed population of bacteria is desirable for a number of applications, such as specific killing of harmful bacteria (4,37). To test this, we designed a targeted conjugation experiment to measure transfer frequencies from a Nb-carrying donor to targeted vs non-targeted recipients over a range of dilutions of the targeted recipient (with non-targeted recipient remaining constant). The set up consisted on 1:1 mixture of donor cells (carrying RP4 and SAtir) with non-target recipient cells. To these matings, we added decreasing proportions (from 1:1 to 1:10 000) of target recipient cells (expressing the Ag TirM on their surface) and measured conjugation frequencies to target and non-target recipient on agar and in liquid broth mixtures (Figure 5 and Supplementary Figure S5).

Regardless of donor bacteria carrying a target-specific adhesin (SAtir) or a control adhesin (SAgfp). Transfer to non-target recipient cell (off-recipients) remained invariable in solid and liquid media (Supplementary Figure S7A). On a solid surface, significant enhancement of conjugation to target recipient cells (expressing the Ag TirM) occurred only when donor cells expressing SAtir were used and the recipient target were mixed at a 1:10 ratio with non-target recipient cells (Supplementary Figure S7B). Liquid conjugation to target recipient cells at a 1:1 ratio significantly increased 2 logs when SAtir donor cells were used instead control donor expressing SAgfp (Figure 5). At least 1-log increase in conjugation frequency to target recipients was remarkably maintained when target recipient were diluted 1:10, 1:100, 1:1000 and even 1:10 000. At this target:non-target ratio, conjugation events per target recipient were close to 1. Therefore, our synthetic adhesion system is not affected by target dilution and elicits a robust transfer of DNA to target recipient cells even when they represent only the 0.01% of the population.

### Targeting conjugation of bacteria expressing EHEC intimin protein

During infection, EHEC bacteria translocate the Tir protein into the intestinal epithelial cells using a type III secretion system (T3SS) (38,39). Tir inserts into the host plasma cell membrane exposing the TirM domain on the surface of the epithelial cells, which acts as a specific receptor for EHEC attachment. The TirM domain binds to intimin C-terminal domains exposed on the bacterial surface (40,41). Intimin-Tir interaction mediates the attachment of EHEC bacteria to the epithelial cells in the intestine, triggering F-actin polymerization in the cytosol of the infected cell underneath the attached bacterium (42). We wondered whether EHEC intimin, as a natural *E. coli* surface-exposed protein Ag, could be used for targeted conjugation.

**Figure 6.**
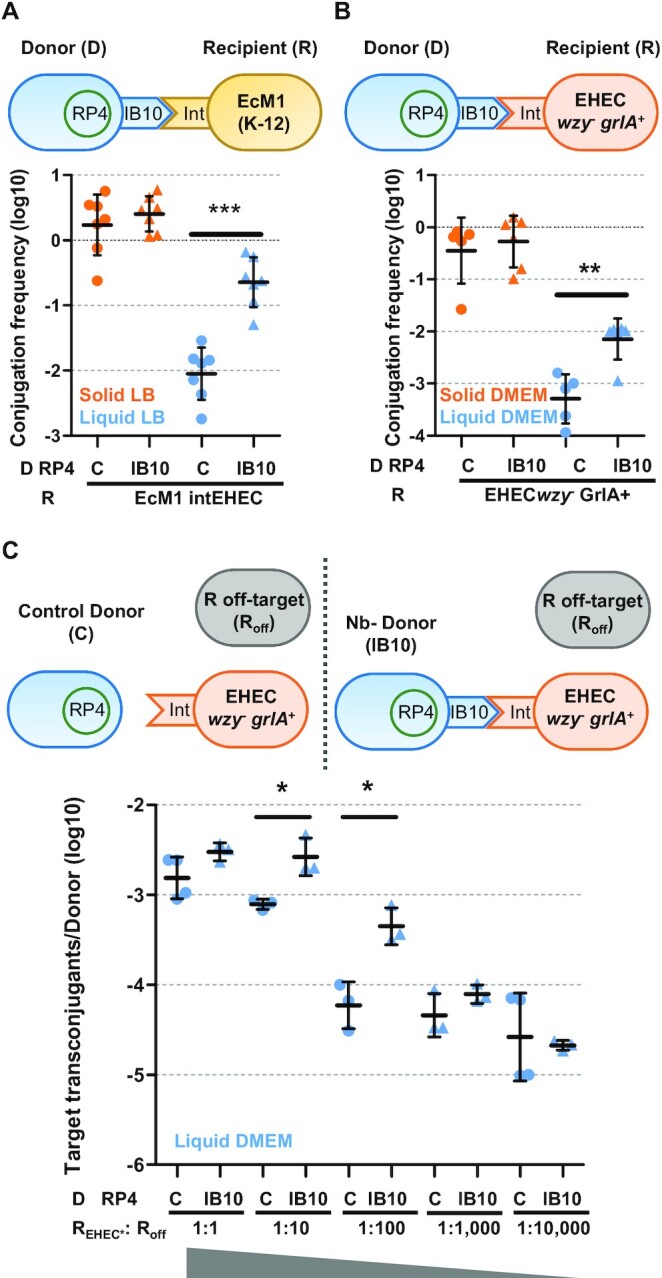
Targeted conjugation to EHEC bacteria with surface-accessible intimin. Mating schemes and conjugation frequencies (Transconjugants/Donor) of RP4 shown in logarithmic scale after grown 2 h in the indicated media. Donor (D) cells were *E. coli* BW27783 carrying RP4 plus either control plasmid pNVgpf (C) or pNeae-IB10 (IB10). Recipients were EcM1_NL-intEHEC in (**A**), EHEC*wzy*^−^*grlA*^+^ in (**B**) and a mixture of target EHEC *wzy*^−^*grlA*^+^ (R_EHEC*_), and off-target BW27783 cells (R_off_) at the indicated R_EHEC*_:R_off_ recipient ratios in (C). *P < 0.05, **P < 0.01, ***P < 0.001, by unpaired Student's *t*-test.

To target intimin, we took advantage of a previously reported Nb (called IB10) that binds to the surface-exposed C-terminal fragment of EHEC intimin (int280_EHEC_) (25,43). This fragment is absent in the N-terminal region of intimin (Neae) used for Nb display. Therefore, Nb IB10 does not bind to synthetic adhesins but recognizes full-length EHEC intimin. With this aim, we constructed a plasmid displaying Nb IB10 (pNeae-IB10; Supplementary Table S2) and found that *E. coli* donor cells carrying RP4 and displaying Nb IB10 efficiently conjugated to *E. coli* recipient cells producing the full-length EHEC intimin (strain EcM1-NL-intEHEC; Supplementary Table S1). In liquid, matings of bacteria with Nb–Ag (IB10-intimin) pairs aggregated (Supplementary Figure S8) and showed conjugation frequencies that significantly exceeded ∼1.5-log that of the control pairs (Figure 6A).

### Targeted conjugation to EHEC bacteria with surface-accessible intimin

Next we used a wild-type strain of EHEC as recipient bacteria in conjugation assays with identical *E. coli* donor cells having RP4 and displaying Nb IB10, as above, but neither cell aggregation nor conjugation enhancement were observed even under growth conditions used for *in vitro* infections in which intimin should be expressed (37 °C, DMEM media and 5% CO_2_; Supplementary Figures S8A and S9A). We hypothesized that thick bacterial surface structures (e.g. LPS and/or capsule) present in EHEC could interfere with the accessibility of intimin for Nb IB10 binding. EHEC produces a group-4 capsule (G4C) made of the same repeating units that its LPS O-antigen.

It is reported that G4C hinders bacterial attachment to host epithelial cells and masks intimin at early time of infection (44). G4C assembly is dependent on *etp*, *etk* and *wzy* genes. Absence of Etk or Etp leads to EHEC cells deficient in G4C formation, but still express LPS O-side chains. However, a *wzy* mutant lacks both LPS O-side chains and the G4C. Therefore, we evaluated bacterial adhesion by mixing *E. coli* BW27783 (RP4+) donor cells displaying Nb IB10 (pNeae-IB10) or control (pNVgfp) with EHEC wild-type or *etk*, *etp*, and *wzy* isogenic mutants. In addition, since the natural expression levels of intimin in EHEC may be low for formation of bacterial adhesion aggregates, we transformed wt and mutant EHEC strains with a plasmid expressing GrlA, the global positive regulator of the *LEE*, which unregulates intimin gene (*eae*) (45). Lastly, aggregation of *E. coli* K-12 strain expressing EHEC intimin (EcM1-NL-intEHEC) was used as a positive control. Quantification of the OD_600_ in the upper part of the co-cultures showed that only donor cells with Nb IB10 x EHEC *wzy*^−^*grlA*^+^ mixtures aggregated to a similar extent than control donor cells IB10 x EcM1-NL-intEHEC pairs (Supplementary Figure S8). Concomitantly, conjugation frequencies of RP4 to EHEC *wzy*^−^*grlA* + strain significantly increased 1-log in liquid matings when donor cells expressed Nb IB10 in comparison to those donors displaying a control Nb binding GFP (Figure 6B).

Besides, we checked whether the targeted conjugation method could be used with EHEC cells in which intimin had been unshielded. In these matings, we added decreasing proportions of EHEC *wzy*^−^*grlA*^+^ target recipient cells together with off-target recipient *E. coli* K-12 cells (Figure 6C). *E. coli* BW27783 (RP4+) displaying Nb IB10 or control SAgfp were used as donor cells, as above. Conjugation on liquid DMEM to prey uncapsulated EHEC were higher at most dilutions when donor cells carried Nb IB10, especially at 1:10 and 1:100, with significant 0.5 and 1-log increases, respectively (Figure 6C). On solid surfaces, conjugation frequencies to EHEC *wzy*^−^*grlA*^+^ mixed at 1:100 with off-target recipient cells were also ∼1-log significantly higher when donor cells displayed Nb IB10 with respect to controls (Supplementary Figure S9B). The enhancing effect was less noticeable at higher dilutions, probably because of a limited Nb–Ag interaction at low concentrations. In any case, this experiment expose the potential of synthetic adhesins to specifically target antigens exposed on the surface of pathogenic bacterial strains to increase transference of a DNA cargo by bacterial conjugation.

## DISCUSSION

The results presented in this work outline how conjugation can be converted to a target-driven DNA delivery device. Our results have both theoretical and practical significance. On the theoretical side, we demonstrate that plasmids that preferably mate on solid media are, in fact, limited by the strength of their bonds within mating pairs in broth. This limitation can be overcome in two basic scenarios. Some plasmids (such as F-like and I-like plasmids) naturally produce their own aggregation factor, which allows them to mate in liquid media, although at the expense of a narrower host range. We demonstrate this point here with the plasmid R1drd19, which can even enhance conjugation of co-resident surface mating plasmids. An alternative with a broader scope of applications may be to use synthetic cell adhesion bridges, such as those we propose here. Synthetic adhesins based on Nb–Ag interaction can drive the specific attachment of *E. coli* bacteria displaying the Nb to Ag-coated surfaces, mammalian cells expressing a surface Ag (15) and other *E. coli* bacteria displaying a cognate Ag on the surface (21,22). The affinity-based conjugation system that uses Nb–Ag pairs is also compatible with synthetic biology applications and will enable design and upscale of efficient liquid conjugation and mobilization events. Synthetic adhesins are efficient, specific, composable and scalable conjugation enhancers, more suitable for biotechnological applications than plasmid-encoded flexible pili. The use of synthetic adhesins increased the transfer of several plasmids 1–3 logs in broth, thus allowing targeted conjugation and transfer of a specific cargo DNA from an engineered donor to a targeted recipient bacterium expressing a surface Ag. This represents a breakthrough achievement for the consecution of specific killing of pathogenic bacteria in complex bacterial communities (4,5,37).

Conjugative transfer is involved in bacterial adaptation to diverse niches through dissemination of accessory genes conferring new abilities to clinical or industrially relevant microorganisms (e.g. antibiotic resistance, stress tolerance …) due to its broad host range. It has practical advantages compared to other natural DNA-delivery methods, such as transformation or transduction, because it is less strain-restricted and less prone to mutations or attacks by the host-cell immune system. However, conjugation, when used as a tool in synthetic biology, is limited by the lack of specificity for the recipient cells, and for the lower yields usually obtained in liquid compared to surface environments. Delivery of therapeutics (e.g. immune response effectors or pathogen killing agents) to the human microbiota of the gastrointestinal tract is among the most important clinical applications involving engineering of indigenous bacteria (46,47). Targeted delivery strategies aim to prevent emerging risks to human health, such as intestinal dysbiosis, or the spread of multiple-drug-resistant bacteria. New, alternative therapies, based on bacteriocins, targeted toxins (4), and especially CRISPR-Cas systems (5,48) also use conjugative DNA transfer as delivery systems. However, in order for these technologies to work efficiently, they must result in high transfer rates to the targeted bacteria.

Although matings have a simpler implementation and are easier to upscale in liquid rather than in solid surfaces, conjugation frequencies of broad host range plasmids encoding rigid pili (e.g. P-pilus) are 2–4 logs lower in cell suspension. Therefore, plasmid transfer is often experimentally quantified at high cell concentration in surfaces, hampering scaling-up conjugation assays by e.g. robotic platforms. This experimental set-up, in which donor and recipient cells form mixed and confluent layers, constrains cell mobility increasing the probability of donor cells to be surrounded by potential recipient cells and increases the conjugation frequencies (13). Therefore, several approaches that already tackled genetic modification of wild microbial communities by conjugation were optimized *in vitro* by conjugation for long periods (3–10 h) over solid surfaces to ensure cell-cell contact (2–4,37). However, plasmid transfer efficiency decreases when the matings are performed either in water or under *in vivo* conditions (up to 300 fold) (4). Therefore, several authors claim that delivery efficiency is the limiting factor in conjugation-based microbial engineering (4,37). Enhancing conjugation frequencies in liquid to target recipients using synthetic adhesins could be very relevant for strategies of microbiome engineering in the gastrointestinal tract. Along the lumen of the gastrointestinal tract, the liquid content is highly variable with diet and other factors influencing water absorption (49), but it is generally high in the stomach and small intestine and progressively lower in the colon and rectum (50). The intestinal epithelium facing the lumen is covered by a gel-like mucus layer of secreted mucins whose composition and integrity is compromised under disease conditions (i.e. inflammation, infection, cancer…) (51). Bacteria of the microbiome are highly abundant in the lumen and the outer layer of mucins, which are environments rich in liquid content and nutrients (52). Further, an increase of the gastrointestinal liquid content occurs during infection and inflammatory bowel diseases, in which there is a reduction in water absorption that leads to diarrhea (50,53).

Synthetic adhesins are efficient conjugation enhancers for a set of prototype plasmids and under different conjugation schemes (Table 1). The highest increase in transfer frequency was obtained in mobilization experiments, where synthetic adhesins improved conjugation up to 3 logs respect to control matings that lack Nb–Ag pairs. The overall effect was dependent on the mating scheme and several different factors. In experiments where RSF1010 mobilization was contingent on the previous conjugation of RP4, the basal frequencies obtained in the absence of synthetic adhesins were low (10^−5^–10^−6^). In these cases, RSF1010 transfer only occurred in the subset of cells that, in the time frame of the experiment, received RP4 and synthetized the conjugation machinery needed to mobilize RSF1010. Both RP4 conjugation and RSF1010 mobilization are favored in the presence of compatible Nb–Ag pairs, thus leading to a large improvement in the overall mobilization frequency. Another likely mechanism by which Nb–Ag pairs may improve the overall RSF1010 mobilization frequency is by overcoming entry exclusion (eex) systems. Experiments showed that Nb–Ag pairs were able to increase the mobilization frequencies even in those experiments where recipients contained an eex expressing plasmid. In triparental mating experiments, the conjugative plasmid may directly enter into the final recipient population, rendering a fraction of it incapable of receiving RSF1010 through entry exclusion. By overcoming this effect, Nb–Ag pairs may also contribute to the overall improvement of the mobilization frequency.

Eex protects recipient cells both from excessive DNA transfer and from cell death by lethal zygosis. Besides, it prevents wasteful plasmid conjugation and inter-plasmid competition (34). The eex mechanism remains unknown, however, in light of our results is tempting to speculate that relies on controlling specific donor-recipient cell contacts needed for conjugation. We can speculate that the strength of the synthetic bridge is such that it overcomes eex almost completely, either by preventing conjugative pilus disassembly or by preserving the mating pair while the system reassembles. Another plausible explanation is that mating pair stabilization by the synthetic bridge increases the probability of newly-synthesized pilus to interact with recipient cells. In any case, the ability of the synthetic adhesion system to partially bypass the eex effect is consistent with its potential use as effective and robust conjugation mediator, avoiding competition with identical plasmid backbones present in potential target pathogens.

The Achilles’ heel of synthetic conjugation approaches as carriers of antimicrobials or other compounds in complex communities is the lack of specificity in the delivery of plasmids to target cells. Even with an improved transfer efficiency and a specific method to exert the effect on the community (e.g. CRISPR-Cas), DNA delivery to non-target bacteria remains an issue (5). T4SSs pili, together with plasmid-encoded natural adhesins (54), are involved in D−R cell contact, resulting in specific cell types being better natural recipients (7). As a result, TraN proteins in F-like plasmids limit the host range of each specific plasmid. Our results, on the other hand, expand the panoply of suitable recipients, by making any selected recipient in a unique target for conjugation. Thus, our method labels target cells to convert conjugation into a specific event. Synthetic adhesion even allows an enriched conjugation on solid media at equal target:off-target proportions or low prey dilutions, suggesting that recipient cells cannot escape from solid conjugators surrounding them. However, spread of rigid-pilus plasmids in liquid is almost restricted to targeted cells, preventing plasmid movement to 99% of the cell population beyond the initial recipient. Therefore, the lower rates of plasmid transfer to undesired bacteria in liquid ensures plasmid contention. When targeted bacteria are diluted in the population, conjugation frequencies are low even when increasing the D/R ratio (4,37). In our liquid experiments, conjugation frequencies to target bacteria were ∼1-log significantly higher even when they were diluted 1:10,000 with non-target cells, anticipating a low frequency of escaping recipients that do not receive the plasmid. Our system thus could optimize the efficiency of genetic weapons and can be combined with existing tools to minimize the appearance of resistant bacteria (4,5).

Our work also provides a proof-of-concept that targeted conjugation could be extended to pathogenic bacteria, such as clinically-relevant EHEC strains. We demonstrate targeted conjugation of EHEC bacteria using synthetic adhesins with a Nb (IB10) binding the surface-exposed C-terminal domains (int280) of intimin (43). Interestingly, our experiments unveiled that an efficient targeted conjugation to EHEC using intimin as Ag requires unshielding the bacterial surface by eliminating LPS and capsule O-polysaccharides (*wzy* mutant) and sufficient expression of the surface Ag (upregulation of intimin by expression of GlrA). These results expose the importance of choosing Ags for the binding of the Nb domain of the synthetic adhesin that are surface-exposed, accessible and well-expressed by the target bacteria. These are critical factors that should be carefully assessed in future strategies aimed to target clinical and/or environmental relevant bacterial strains in their natural niches. Given the versatility of *E. coli* display of the selection of high-affinity Nbs against any potential Ag of interest (20), identifying the appropriated surface-exposed Ag on the target bacterial strain will allow the development of effective synthetic adhesins for targeted conjugation in natural environments. These Ags may include abundant outer membrane proteins (e.g. porins, adhesins), fimbrial subunits, or extracellular polysacharides, such as surface-exposed O-antigen (55), and even polysacharides found in biofilms (56,57). Nbs selected against these Ags may allow attachment of the donor bacteria with the synthetic adhesin in direct contact (or in close proximity) to the target recipient cell to facilitate conjugation. In conclusion, this work shows the potential of synthetic adhesins in combination with bacterial conjugation for the selective delivery of cargo DNAs to target recipients in complex bacterial populations.

## DATA AVAILABILITY

All data are available in the main text or the supplementary materials.

## Supplementary Material

gkac1164_Supplemental_File
