# Supplementary material for: Targeted bacterial conjugation mediated by synthetic cell-to-cell adhesions
Source: Nucleic Acids Res. 2022 Dec 13;50(22):12938–50. doi: 10.1093/nar/gkac1164 (PMC9825185; doi:10.1093/nar/gkac1164)
Supplement: gkac1164_Supplemental_File [file gkac1164_supplemental_file.pdf]

Supplementary Materials for

Targeted bacterial conjugation mediated by synthetic cell-to-cell adhesions

Marta Robledo[1,2]*, Beatriz Álvarez[3], Ana Cuevas[1], Sheila González[1], David Ruano-Gallego[3], Luis Ángel Fernández[3] and Fernando de la Cruz[1]*

[1]Intergenomics Group, Instituto de Biomedicina y Biotecnología de Cantabria, Universidad de Cantabria, Santander, Cantabria, 39011, Spain.
[2]Biomar Microbial Technologies, Parque Tecnológico de León, Armunia, León, 24009, Spain.
[3]Department of Microbial Biotechnology, Centro Nacional de Biotecnología, Consejo Superior de Investigaciones Científicas (CNB-CSIC), Campus UAM Cantoblanco, 28049, Madrid, Spain

* To whom correspondence should be addressed. Email: delacruz@unican.es
Correspondence may also be addressed to marta.robledo@unican.es

This PDF file includes:

Supplementary Figures S1 to S9
Supplementary Tables S1 to S4

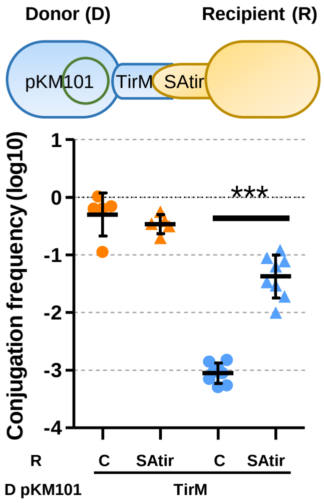

Fig. S1. Synthetic adhesion increase conjugation frequencies in liquid media of conjugative plasmid pKM101. Experimental scheme and conjugation frequencies (Transconjugants/Donor) of pKM101 shown in logarithmic scale after grown 2h in either solid or in liquid LB medium. Donor (D) bacteria were EcM1flu::TirMA, constitutively expressing TirM Ag on the bacterial surface. Recipient (R) cells were *E.coli* BW27783 carrying either pNVgfp (Control; C) or pNVtir1 (SAtir). Each point represents the result of one independent experiment shown in logarithmic scale; horizontal and vertical bars represent the mean ± SD of each group of data. ***$p < 0.001$, by unpaired Student's t-test.

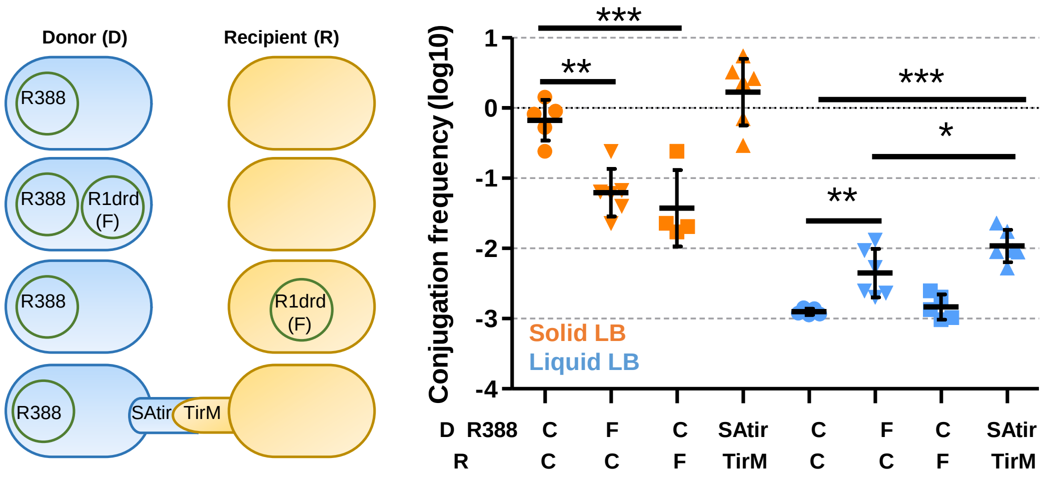

Fig. S2. Synthetic adhesins are more efficient conjugation enhancers than IncF liquid-mating plasmids. Conjugation schemes and frequencies (transconjugants/donor = T/D) of conjugative plasmid R388 shown in logarithmic scale after grown 2h in either solid or in liquid LB. Donor carried R388 alone (Control; C) or plus either the IncF liquid conjugator R1drd (F) or pNVtir (SAatir), as indicated. Recipient cells were either plasmid-free *E. coli* MG1655 EcM1 (Control; C), EcM1 carrying R1drd (F) or EcM1flu::TirMA (TirM), as depicted in the scheme. *$p < 0.05$, **$p < 0.01$, ***$p < 0.001$, by unpaired Student's t-test.

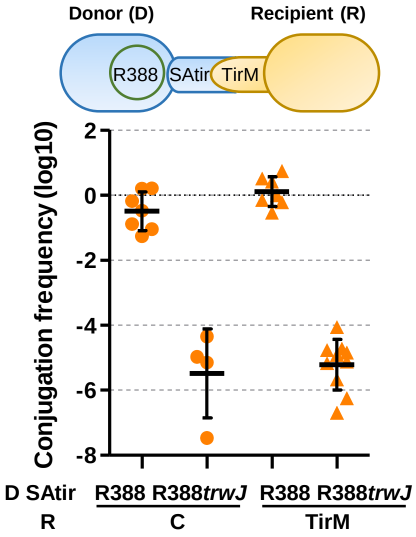

Fig. S3. Synthetic adhesion does not complement lack of pili adhesin for conjugation. Experimental scheme and conjugation frequencies (Transconjugants/Donor) of plasmids R388 and R388 *trwJ* shown in logarithmic scale after grown 2h in either solid or in liquid LB medium. Donor bacteria were *E. coli* BW27783 carrying either R388 or R388 *trwJ* derivative plus pNVtir1, expressing tir1 anti-TirM Nb. Recipient cells were either *E. coli* MG1655 EcM1 (Control; C) or EcM1flu::TirMA (TirM). Each point represents the result of one independent experiment shown in logarithmic scale; horizontal and vertical bars represent the mean ± SD of each group of data.

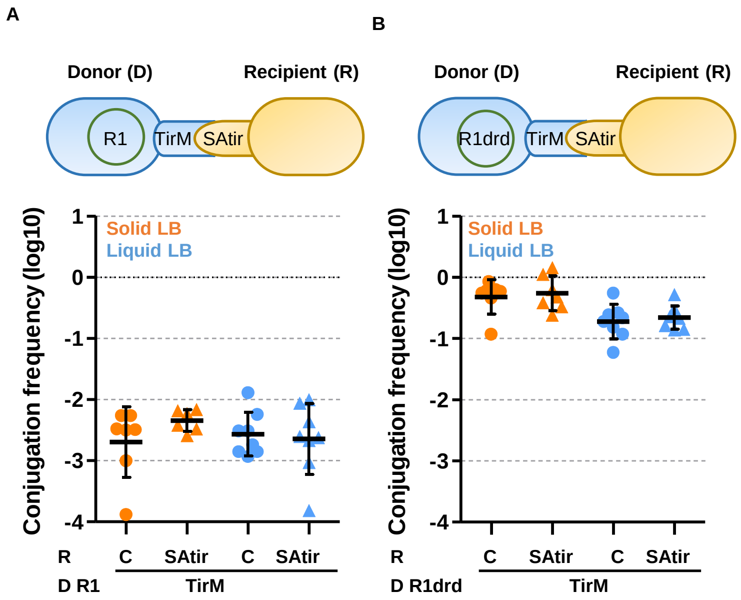

Fig. S4. Synthetic adhesion of donor-to-recipient cells does not affect conjugation of liquid-mating plasmids. Conjugation schemes and frequencies (transconjugants/donor = T/D) of low-frequency conjugative plasmid R1 **(A)** and high-frequency R1drd19 **(B)** shown in logarithmic scale after grown 2h in either solid or in liquid LB. Donor were EcM1flu::TirMA, constitutively expressing TirM peptide carrying the corresponding conjugative plasmid. Recipients carried either control pNVgpf (Control; C) or pNVtir1 (SAtir).

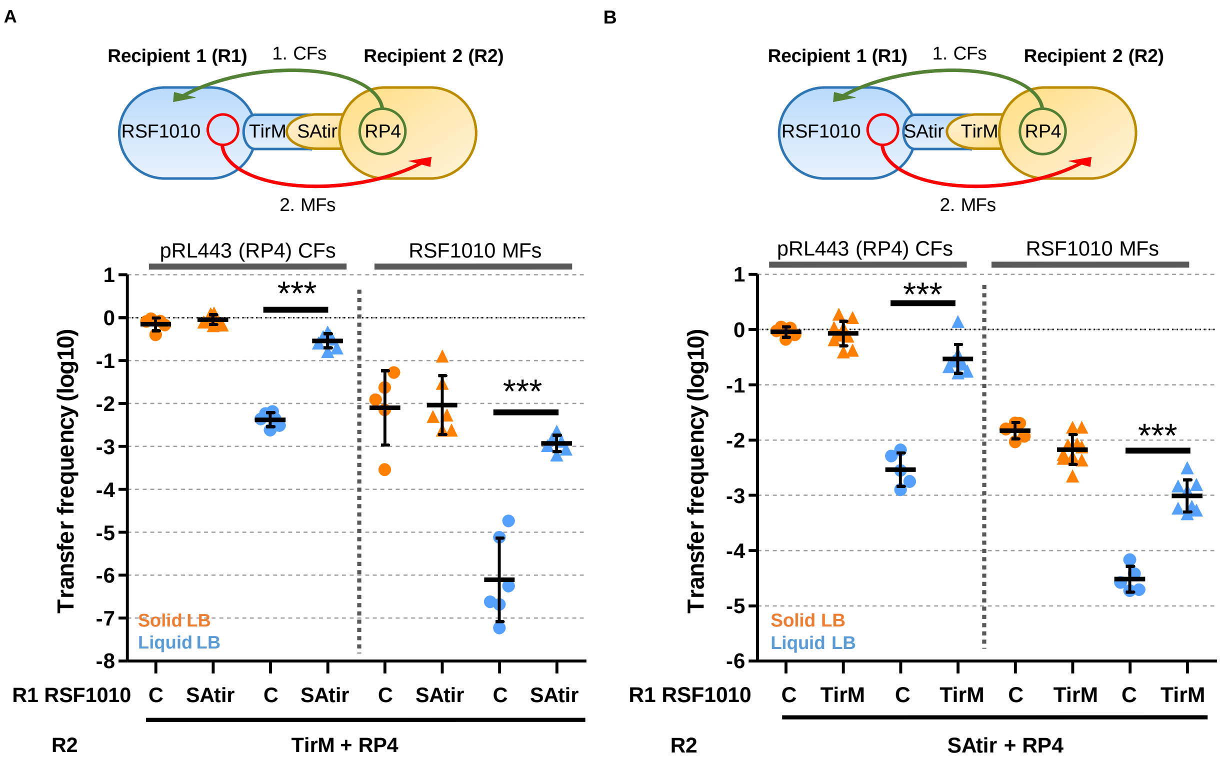

Fig. S5. Synthetic adhesion increases RP4-mediated RSF1010 mobilization frequencies. RP4 conjugation frequencies (CFs; transconjugants/recipient 1 T/R1) and RSF1010 mobilization frequencies (MFs; T/R2), in depicted matings shown in logarithmic scale after grown 2h in either solid (orange points) or liquid (blue) LB medium. **(A)** R1 bacteria were *E. coli* BW27783 carrying the mobilizable plasmid RSF1010 plus either control plasmid pHEA (Control; C) or pTirMA (TirM) expressing the TirM Ag. R2 cells were EcM1SAtir, constitutively expressing the Nb (SAtir) and carrying conjugative plasmid RP4. **(B)** R1 bacteria were *E. coli* BW27783 carrying the mobilizable plasmid RSF1010 plus either control plasmid pNVgfp (Control; C) or pNVtir1 (SAtir). R2 cells were EcM1TirMA, constitutively expressing the Ag TirM and carrying conjugative plasmid RP4.

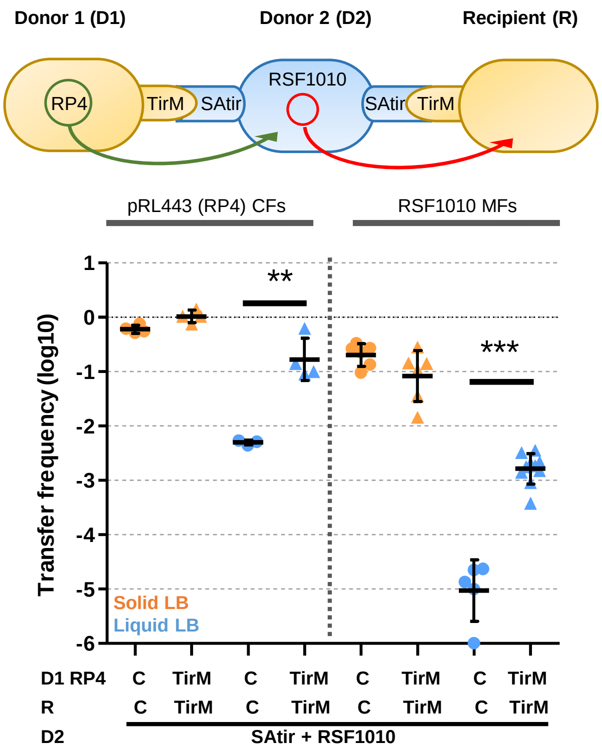

Fig. S6. Synthetic adhesion enhances DNA transference in complex triparental mating schemes. Triparental mattings: Donor 1 (D1) and recipient (R) bacteria were *E. coli* BW27783 carrying either pHEA (Control; C) or pTirMA (TirM). Donor 2 (D2) cells were *E. coli* MG1655 EcM1SAtir expressing the tir Nb (SAtir) and carrying the mobilizable plasmid RSF1010. D1 cells also carried RP4 plasmid, which mobilizes RSF1010 upon conjugation to D2 cells. $^{**}p < 0.01$, $^{***}p < 0.001$, by unpaired Student's t-test.

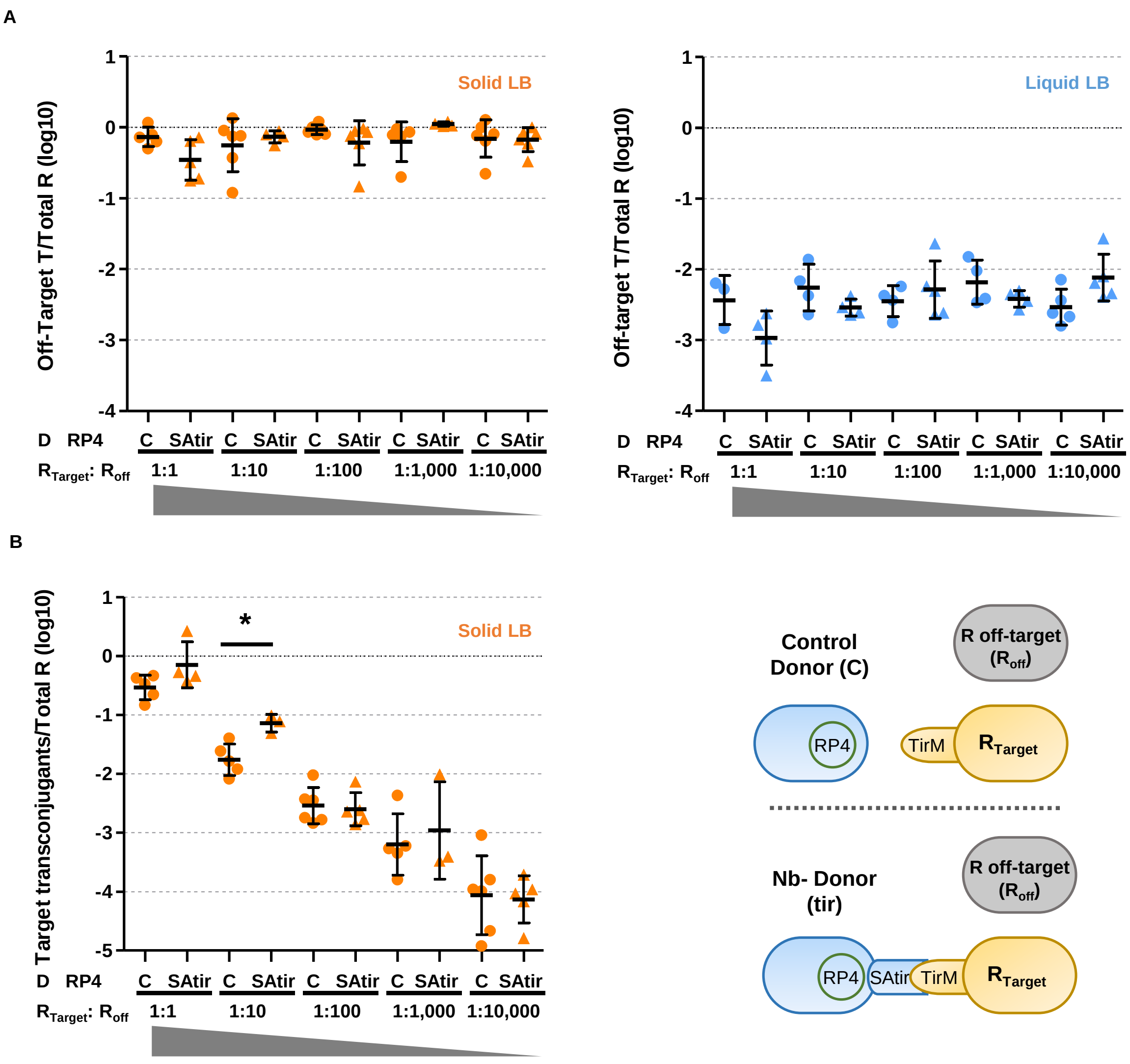

Fig. S7. Conjugation to non-target (A) and target recipient cells in solid surfaces (B). Conjugation frequencies of RP4 (IncP) expressed as log10 of off-target **(A)** or target **(B)** transconjugants per total number of recipients after grown 2h in solid or liquid LB medium (target transconjugants per total number of recipients in liquid LB medium is shown in figure 5). Overview of the target conjugation control and experiments with compatible SAtir/TirM expressing Donor and R_{target} bacteria is also shown. Donor were *E. coli* BW27783 cells carrying plasmid RP4 and either pNVgpf (Control; C) or pNVtir1 (SAtir), expressing the anti-TirM Nb upon induction with 0.1 mM IPTG. Recipient cells were a mixture of target EcM1flu::TirMA cells (R_{Target}), constitutively expressing TirM peptide and non-target BWmKate cells (R off-target; R_{off}). Donor, TirM target and control recipient cells were previously grown overnight without shaking and mixed at the indicated R_{Target}:R_{off} recipient ratios, decreasing the proportion of R_{Target} cells (Donor and $R_{off\text{-}target}$ cells were always at a 1:1 proportion). *$p < 0.05$, by unpaired Student's t-test.

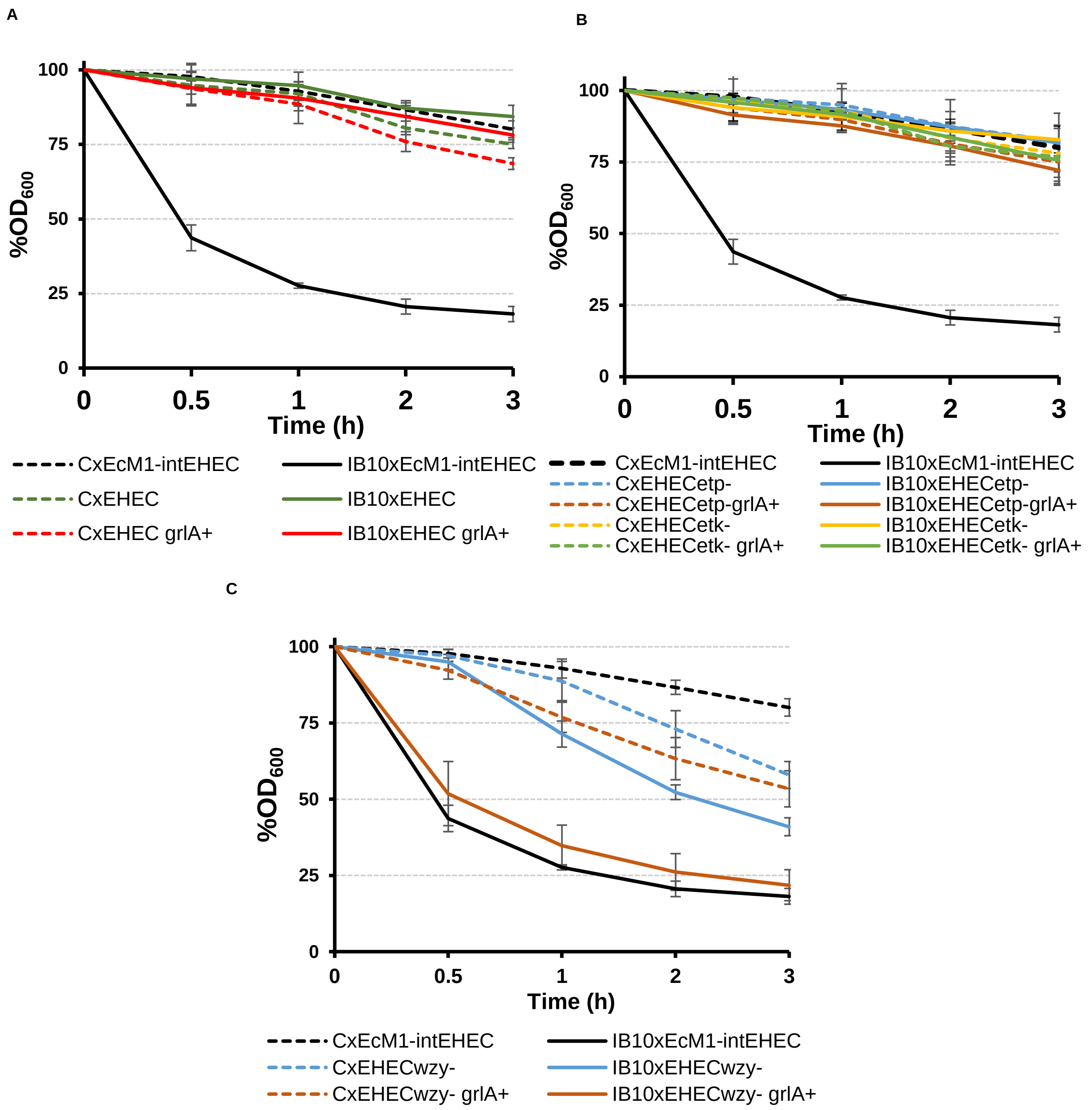

Fig. S8. Mixtures of IB10-expressing cells and EHEC *wzy*$^{-}$ *grlA*$^{+}$ aggregated to a similar extent than with EcM1-NL-intEHEC. Shown is relative OD_{600} at different time points of cells remaining at upper part of liquid matings. *E. coli* BW27783 carrying RP4 and either plasmid pNVgpf (Control; C) or pNeae-IB10 (IB10), plus either EcM1-NL-intEHEC, constitutively expressing the full-length Intimin from EHEC **(A)** or the indicated EHEC mutant strain: *etp*$^{-}$, *etk*$^{-}$ **(B)** or *wzy*- **(C)** with or without carrying pSA10_GrlA-6his to express GrlA (GrlA^{+}) upon addition of 0.02 mM IPTG.

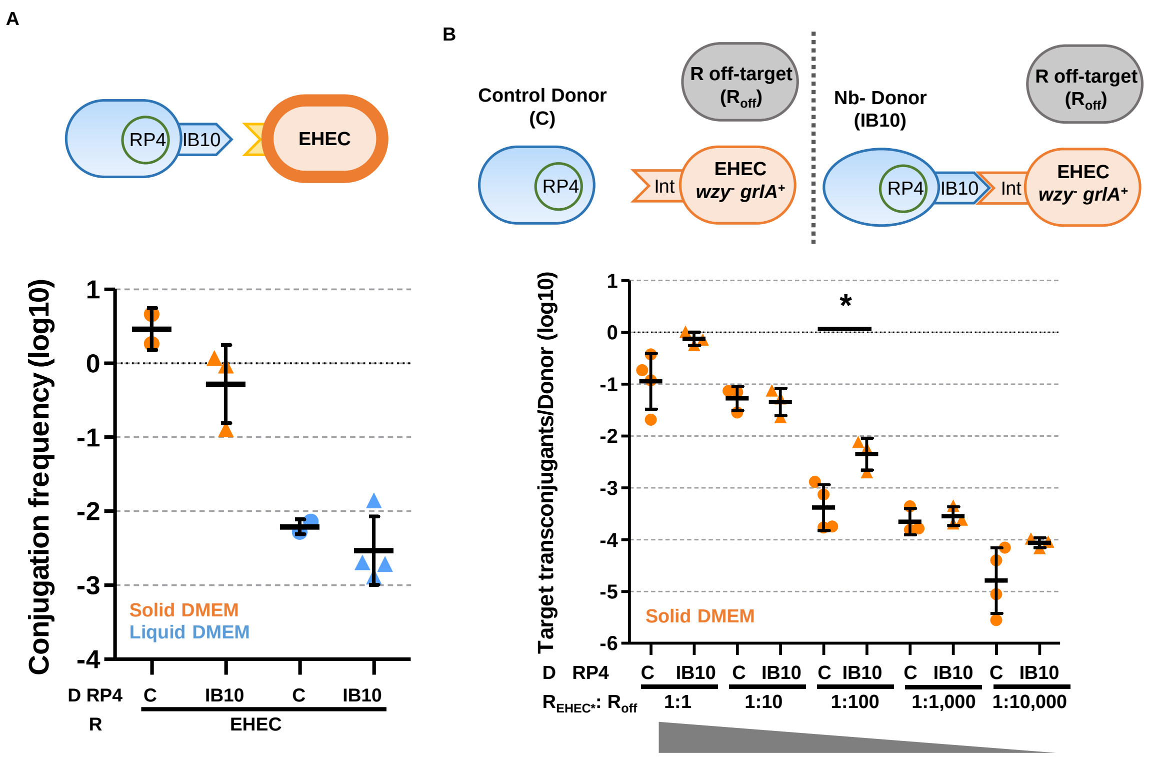

Fig. S9. IB10-expressing donors do not increase CFs to wt capsulated EHEC (A) but allow targeted conjugation to uncapsulated EHEC on solid surfaces (B). Mating schemes and CFs (T/D) of plasmid RP4 shown in logarithmic scale after grown 2 h in DMEM media at 37 °C and 5% CO_2. **(A)** Donor were carrying RP4 plus either pNVgpf (Control; C) or pNeae-IB10 (IB10). Recipients were wild type EHEC. **(B)** Donor were *E. coli* BW27783 cells carrying RP4 and either pNVgpf (Control; C) or pNeae-IB10 (IB10), expressing the IB10 Nb anti-Intimin (from EHEC) upon induction with 0.1 mM IPTG. Recipient cells were a mixture of target EHEC *wzy⁻ grlA⁺* (R_{EHEC*}), and off-target BW27783 cells (R_{off}). Donor, EHEC and non-target recipient cells were previously grown overnight without shaking and mixed at the indicated R_{EHEC}:R_{off} recipient ratios, decreasing the proportion of R_{EHEC*} cells (donor and non-target control cells were always at a 1:1 proportion). *$p < 0.05$, by unpaired Student's t-test.

Table S1. ***E. coli*** **strains used in this study**

Name	Relevant Characteristics	Source
DH10B-T1R	(F- λ-) *mcrA* Δ*mrr-hsdRMS-mcrBC φ80lacZDM15* Δ*lacX74 recA1 endA1 araD139 Δ(ara, leu)7697 galU galK rpsL (StrR) nupG tonA*	Novagen
BW25141	(F- λ-) Δ*(araD-araB)567* Δ*lacZ4787(::rrnB-3)* Δ*(phoB-phoR)580 galU95* Δ*uidA3::pir recA1 endA9(delins)::FRT rph-1,* Δ*(rhaD-rhaB)568 hsdR51*	(*1*)
MG1655	K-12 (F- λ-)	(*2*)
EcM1	MG1655 *ΔfimA-H*	(*3*)
EcM1TirMA	EcM1*Δflu::P_{N25}-TirM*-C-EhaA [pelB-$TirM_{EHEC}$ -E-tag-EhaA(989-1327)]	This work
EcM1SAgfp	EcM1*Δflu::P_{N25}-SAgfp* [IntiminEHEC (1-654)-E-Vgfp-myc-tag]	(*4*)
EcM1SAtir	EcM1*Δflu::P_{N25}-SAtir* [IntiminEHEC (1-654)-E-Vtir1-myc-tag]	(*4*)
EcM1-NL-intEHEC	EcM1 Δ*lacIZ* Δ*flu*::*$lacI^q$*-Ptac-*eae* (intiminEHEC 1-934)-*aac(3)IV* ($Apra^R$)	This work
BW27783	*$lacI^q$ rrnB3* Δ*lacZ4787 hsdR514 Δ(araBAD)56 7Δ(rhaBAD)568*	(*5*)
BWmKate2	BW27783-Nx^R attTn7:: P_{lac}-mKate2. Km^R	R. Fernández-López
EHEC stx^-	EHEC O157:H7 *$stx1^-$ $stx2^-$* mutant of strain EDL933 (TUV93-0)	(*6*)
EHEC *wzy^-*	EDL933 *stx^- wzy*::mini-Tn5kan2 (SK2526)	(*6*)
EHEC *etp^-*	EDL933 stx^- Δ*etp*::*cm* (SK2235)	(*6*)
EHEC *etk^-*	EDL933 stx^- Δ*etk::kan* (SK1416)	(*6*)

Table S2. Plasmids used in this study.

Name	Relevant Characteristics	Source
pAK-Not	Cm^R, pBR322-ori, lacIq-Plac	(*7*)
pNeae2	Cm^R, pAK-Not derivative with intimin Neae fragment [IntiminEHEC (1-654)-E-His-myc-tag]	(*8*)
pNVgfp	Cm^R, pNeae2-derivative with Nb anti-GFP SAgfp [IntiminEHEC (1-654)-E-Vgfp-myc-tag]	(*8*)
pNVtir1	Cm^R, pNeae2-derivative with Nb anti-TirMEHEC SAtir [IntiminEHEC (1-654)-E-Vtir1-myc-tag]	(*8*)
pNeae-IB10	Cm^R, pNeae2-derivative with Nb anti-int280EHEC [IntiminEHEC (1-654)-E-VIB10-myc-tag]	This work
pHEA	Cm^R, pAK-Not derivative; His-tagged C-EhaA [pelB-His-E-tag-EhaA(989-1327)]	(*9*)
pTirMA	Cm^R, pHEA derivative with TirM (residues 250-352 of EHEC Tir) fused to C-EhaA [pelB-TirMEHEC-E-tag-EhaA(989-1327)]	This work
pGE	Km^R; R6K-ori, muticloning site and I-SceI sites	(*4*)
pGEflu	Km^R, pGE with homology regions (HRs) flanking flu gene	(*4*)
pGEfluSAgfp	Km^R, pGEflu with PN25-SAgfp [IntiminEHEC (1-654)-E-Vgfp-myc-tag]	(*4*)
pGEfluSAtir	Km^R, pGEflu with PN25-SAtir [IntiminEHEC (1-654)-E-Vtir1-myc-tag]	(*4*)
pGEfluTirMA	Km^R, pGEflu HRs and PN25-TirMA [IntiminEHEC (1-654)-E-Vtir1-myc-tag]	This work
pGETS	Km^R; pSC101-ts ori, multicloning site and I-SceI sites	(*10*)
pGERecombTS-intEHEC	Km^R, $Apra^R$, pGETS, C-intimin, flu HR, aac(3)IV	This work
pSA10_GrlA-6xhis	Amp^R, pBR322 derivative, Plac-GrlA	(*11*)
pRL443 (RP4)	Conjugative. IncP1α; MOBP11; MPFT. RP4 derivative lacking Km^R gene.	(*12*)

pAR106	Conjugative. IncP1α; RP4 derivative with a istAB-aph insertion carrying gfp.	E. Zechner
pKM101	Conjugative, IncN; MOBF11; MPFT	(*13*)
R388	Conjugative, IncW; MOBF11; MPFT	(*14*)
R388 *trwJ*	R388 carrying a Tn5tac1 insertion mutation in *trwJ* (pSU4136)	(*15*)
R1	Conjugative, IncFII; MOBF12; MPFF	(*12*)
R1drd-19	Conjugative, IncFII; MOBF12; MPFF	(*16*)
RSF1010	Plasmid mobilizable by pRL443 (RP4)	(*17*)

Inc, incompatibility group (*18*). MOB, MOB group (*19*). MPF, mating pair formation (*20*).

Table S3. List of primers used in this study.

Name	Sequence (5'-3')*	Purpose
F_SfiI_TirM	CTCGCGGCCCAGCCGGCCCAGGCGCTTGCATTGACGCC	Cloning of TirM
R_NotI_TirM	CCGCAGCGGCCGCCGATGAAACTTTCAGCTCCTCCTGG	Cloning of TirM
5' XhoI HR	CATTTCTCGAGGGATGATAGTGCATTACGCAGTCAGG	Cloning of C-ter intimin
3' SpeI HindIII int	GCTGAACTAGTAAGCTT TTCTACACAAACCGCATAGACATTTGG	Cloning of C-ter intimin
5' HindIII FRT Apra	CCAGAAGCTTGAAGTTCCTATTCCGAAGTTCCTATTCTCTAGAAAGTATAGGAACTTCTGCAGCTCACGGTAACTGATGCCG	Cloning of apramycin resistant cassette
3'_SpeI_FRT_Apra	GGAAACTAGTGAAGTTCCTATACTTTCTAGAGAATAGGAACTTCGGAATAGGAACTTCGGAATAGGAACTTATGAGCTCAGCC	Cloning of apramycin resistant cassette
VHH-SfiI2	GTCCTCGCAACTGCGGCCCAGCCGGCCATGGCTCAGGTGCAGCTGGTGGA	Cloning of Nb IB10
VHH-NotI2	GGACTAGTGCGGCCGCTGAGGAGACGGTGACCTGGGT	Cloning of Nb IB10
F_flu_int	CGGTTCACAGGCAATTGGCGGTATTGTTAAC	Confirmation of cassette integration in *flu* locus
Int_EHEC_seq	CAGACATCTAGTGAGCAGCGTTCTGG	Confirmation of full-length intimin integration in *flu* locus
3' flu genome	GAAGCTGATGATCATTCCGGCAATC	Confirmation of full-length intimin integration in *flu* locus

Table S4. Tir-anchored CFs of conjugative plasmids RP4 and R388 increase with time.Conjugation frequencies (CFs) are expressed as fold increased relative to control.

					CF Fold increase	
**Plasmid**	**Inc**	**Pilus**	**MOB**	**Time**	**Solid**	**Liquid**
RP4	P1α	Rigid	F11	1 h	1.03	67.40***
				2 h	1.08	151.90***
R388	W	Rigid	F11	1 h	2.40	7.50***
				2 h	4.04	29.74***

Inc, incompatibility group. MOB, MOB group. ***$p < 0.001$, by unpaired Student's t-test.

References

1. K. A. Datsenko, B. L. Wanner, One-step inactivation of chromosomal genes in *Escherichia coli* K-12 using PCR products. *Proc Natl Acad Sci U S A* **97**, 6640-6645 (2000).
2. F. R. Blattner *et al.*, The complete genome sequence of *Escherichia coli* K-12. *Science* **277**, 1453-1462 (1997).
3. I. C. Blomfield, M. S. McClain, B. I. Eisenstein, Type 1 fimbriae mutants of *Escherichia coli* K12: characterization of recognized afimbriate strains and construction of new fim deletion mutants. *Mol Microbiol* **5**, 1439-1445 (1991).
4. C. Piñero-Lambea *et al.*, Programming controlled adhesion of *E. coli* to target surfaces, cells, and tumors with synthetic adhesins. *ACS Synth Biol* **4**, 463-473 (2015).
5. A. Khlebnikov, K. A. Datsenko, T. Skaug, B. L. Wanner, J. D. Keasling, Homogeneous expression of the P(BAD) promoter in *Escherichia coli* by constitutive expression of the low-affinity high-capacity AraE transporter. *Microbiology* **147**, 3241-3247 (2001).
6. Y. Shifrin *et al.*, Transient shielding of intimin and the type III secretion system of enterohemorrhagic and enteropathogenic *Escherichia coli* by a group 4 capsule. *J Bacteriol* **190**, 5063-5074 (2008).
7. E. Veiga, V. de Lorenzo, L. A. Fernández, Probing secretion and translocation of a beta-autotransporter using a reporter single-chain Fv as a cognate passenger domain. *Mol Microbiol* **33**, 1232-1243 (1999).
8. V. Salema *et al.*, Selection of single domain antibodies from immune libraries displayed on the surface of *E. coli* cells with two β-domains of opposite topologies. *PLoS One* **8**, e75126 (2013).
9. E. Marín, G. Bodelón, L. Fernández, Comparative analysis of the biochemical and functional properties of C-terminal domains of autotransporters. *J Bacteriol* **192**, 5588-5602 (2010).
10. D. Ruano-Gallego, B. Álvarez, L. Fernández, Engineering the Controlled Assembly of Filamentous Injectisomes in *E. coli* K-12 for Protein Translocation into Mammalian Cells. *ACS Synth Biol* **4**, 1030-1041 (2015).
11. Y. Litvak *et al.*, Epithelial cells detect functional type III secretion system of enteropathogenic *Escherichia coli* through a novel NF-κB signaling pathway. *PLoS Pathog* **13**, e1006472 (2017).
12. J. Elhai, A. Vepritskiy, A. M. Muro-Pastor, E. Flores, C. P. Wolk, Reduction of conjugal transfer efficiency by three restriction activities of *Anabaena* sp. strain PCC 7120. *J Bacteriol* **179**, 1998-2005 (1997).
13. P. J. Langer, W. G. Shanabruch, G. C. Walker, Functional organization of plasmid pKM101. *Journal of bacteriology* **145**, 1310-1316 (1981).
14. N. Datta, R. W. Hedges, Trimethoprim resistance conferred by W plasmids in Enterobacteriaceae. *Journal of general microbiology* **72**, 349-355 (1972).

15. H. D. de Paz *et al.*, Functional interactions between type IV secretion systems involved in DNA transfer and virulence. *Microbiology (Reading)* **151**, 3505-3516 (2005).
16. E. Meynell, N. Datta, Mutant drug resistant factors of high transmissibility. *Nature* **214**, 885-887 (1967).
17. K. M. Derbyshire, G. Hatfull, N. Willetts, Mobilization of the non-conjugative plasmid RSF1010: a genetic and DNA sequence analysis of the mobilization region. *Mol Gen Genet* **206**, 161-168 (1987).
18. D. E. Taylor, A. Gibreel, T. D. Lawley, D. M. Tracz, in *Plasmid Biology,* B. E. Funnel, G. J. Phillips, Eds. (ASM Press, Washington, DC, 2004), chap. 23, pp. 473-491.
19. M. P. Garcillan-Barcia, M. V. Francia, F. de la Cruz, The diversity of conjugative relaxases and its application in plasmid classification. *FEMS microbiology reviews* **33**, 657-687 (2009).
20. J. Guglielmini, L. Quintais, M. P. Garcillan-Barcia, F. de la Cruz, E. P. Rocha, The repertoire of ICE in prokaryotes underscores the unity, diversity, and ubiquity of conjugation. *PLoS genetics* **7**, e1002222 (2011).
